# Mesenchymal stromal cells as rescue therapy in biologic-refractory psoriasis: insights from a case series

**DOI:** 10.3389/fimmu.2025.1656724

**Published:** 2025-09-03

**Authors:** Su M. Lwin, Shane Solanky, Cristiano Scottà, Chiara Giacomini, Shir Azrielant, Isabella Tosi, Atheer Al-Haddabi, Emelia Duarte-Williamson, Hannah Dawe, Sarah Walsh, John A. McGrath, Giovanna Lombardi, Francesco Dazzi, Paola Di Meglio, Christopher E. M. Griffiths

**Affiliations:** ^1^ St John’s Institute of Dermatology, Guy’s and St Thomas’ NHS Foundation Trust, London, United Kingdom; ^2^ St John’s Institute of Dermatology, School of Basic and Medical Biosciences, King’s College London, London, United Kingdom; ^3^ College of Health, Medicine and Life Sciences, Department of Life Science, Biosciences, Brunel University London, London, United Kingdom; ^4^ Peter Gorer Department of Immunobiology, School of Immunology and Microbial Sciences, King’s College London, London, United Kingdom; ^5^ School of Cardiovascular and Metabolic Medicine & Sciences, King’s College London, London, United Kingdom; ^6^ Division of Dermatology, Tel Aviv Sourasky Medical Center, Tel Aviv, Israel; ^7^ Department of Dermatology, King’s College Hospital, London, United Kingdom; ^8^ King’s Health Partners Centre for Translational Medicine, London, United Kingdom

**Keywords:** psoriasis, multiple biologic-refractory, mesenchymal stromal cells, immunomodulatory, regulatory T cells, monocytes

## Abstract

Cytokine-targeted biologics have revolutionized the management of moderate-to-severe psoriasis; however, all available therapies have failed a growing number of patients. Mesenchymal stromal cells (MSCs), with their immunomodulatory properties, offer a novel therapeutic option. Here, we report the cases of three adult female patients with long-standing, severe plaque psoriasis who were refractory to multiple biologic therapies, and were consequently treated with two intravenous infusions of allogeneic umbilical cord-derived MSCs (UC-MSCs; 1.96 – 3.00 × 10^6^ cells/kg) 1 week (W) apart. Two patients received UC-MSCs as monotherapy; one received them alongside etanercept. Upon relapse, two patients resumed their last failed biologic at W9, while one switched to a new biologic at W24. UC-MSCs were well-tolerated and yielded variable clinical benefits. The best responder to MSCs experienced an 87% reduction in the Psoriasis Area and Severity Index (PASI 87) by W4. Two patients showed improved responses to previously failed biologics (absolute PASI of 0–2), sustained for over 2 years following reinitiation. Multi-parameter flow cytometry revealed increased frequencies of CD4^+^ and CD8^+^ skin-homing (CLA^+^CD103**^−^**) and skin-recirculating (CLA^+^CD103^+^) memory T cells, CD25^Hi^CD127^Lo^FoxP3^+^ regulatory T cells, and non-classical (CD14^Lo^CD16^+^) monocytes, associated with clinical improvements. These findings suggest that UC-MSCs may potentially provide direct benefits for biologic-refractory psoriasis and restore responsiveness to previously ineffective biologics, possibly by resetting the immune response. Further investigation in larger cohorts is warranted.

## Introduction

Psoriasis is a common, debilitating, and life-shortening inflammatory skin disease that affects more than 60 million people worldwide. It presents as red (gray on darker skin), heavily scaled plaques that can appear on any skin surface, severely impacting physical and mental health. The disease usually starts before the age of 30 (1), and is linked to systemic inflammation, including arthritis, heart disease, diabetes, and depression, making it a serious systemic condition. It is caused by gene–environment interactions and a complex interplay of innate and adaptive immunity, resulting in a perpetual inflammatory loop underpinned by the interleukin (IL)-23/type 17 T cell (Th17) axis (1). Biologics that target cytokine pathways have transformed the management of psoriasis, but none are curative (2). Furthermore, all available classes of biologic therapy have failed an increasing number of patients, i.e., tumor necrosis factor (TNF), IL - 17, IL - 23p40, and IL - 23p19 inhibitors (3, 4). Mesenchymal stromal cells (MSCs) are a heterogeneous population of multipotent stem cells with immunomodulatory properties and low immunogenicity, which renders them an attractive form of cell therapy for immune-mediated inflammatory diseases (IMIDs) (5–8). In recent years, case series and small clinical trials have reported on the safety and efficacy of MSCs in patients with psoriasis (9–14). However, to date, studies on the use of MSCs in the difficult-to-treat, multiple-biologic-failure psoriasis population are sparse (5, 13, 14). We report on the safety, efficacy, and immunomodulatory capacity of systemic allogeneic umbilical cord-derived MSCs (UC-MSCs) in three adults with severe, biologic-refractory psoriasis over 2 years of follow-up.

## Case descriptions

### Patient 1

**Figure 1:**
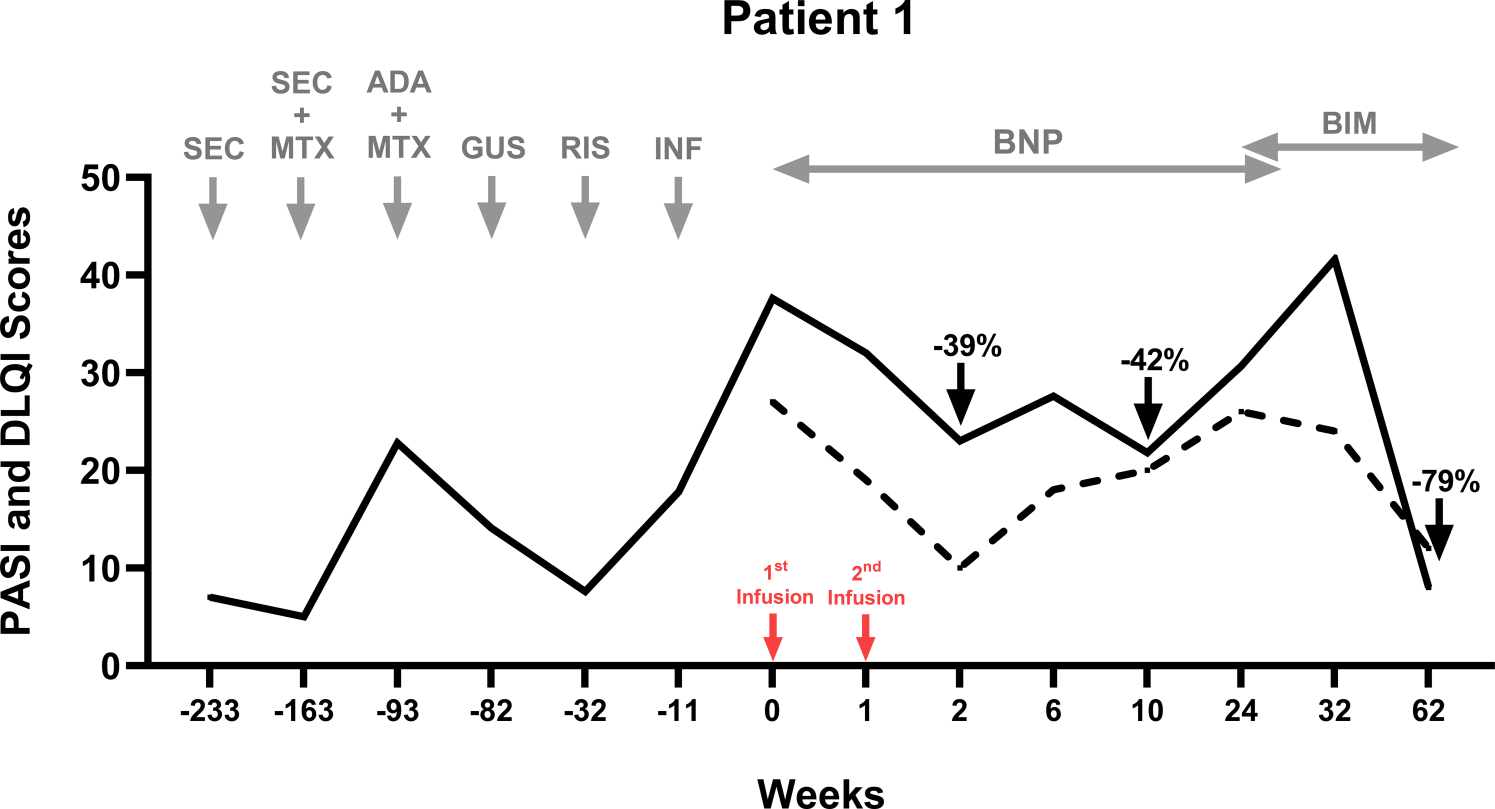
Therapeutic timeline of Patient 1. Six biologics across three classes failed in treating Patient 1. UC-MSCs were administered as an adjuvant therapy alongside Benepali (an etanercept biosimilar). Benepali was initiated 9 weeks prior to the UC-MSC infusions. The PASI score (black line) and the DLQI score (dotted line) improved after two infusions at Weeks 0 and 1 (red arrows). Benepali was switched to a new biologic—bimekizumab—at Week 24, when psoriasis flared and the PASI returned to baseline score. Patient 1 experienced a reduction in the PASI by 39% at Week 2 and 42% at Week 10 post-MSCs, and by 79% 38 weeks after the initiation of bimekizumab. ADA, adalimumab; BIM, bimikizumab; BNP, Benepali (etanercept biosimilar); GUS, guselkumab; INF, infliximab; MTX, methotrexate; RIS, risankizumab; and SEC, secukinumab.

A 46-year-old white European woman with plaque psoriasis for 25 years presented with a severe flare of the disease while on an etanercept biosimilar (Benepali, anti-TNF) (**Figure 1**). Benepali was initiated following an infusion reaction to infliximab. She had secondary failure to five biologics across four classes—secukinumab (anti-IL-17A; ± methotrexate), adalimumab (anti-TNF; ± methotrexate), guselkumab (anti-IL-23p19), risankizumab (anti-IL23p19), and infliximab (anti-TNF)—and had primary failure to ixekizumab (anti-IL-17A). Comorbidities included psoriatic arthritis, type 2 diabetes, non-alcoholic fatty liver disease (NAFLD), anxiety, and depression. Examination revealed large, widespread, inflammatory plaques involving high-impact sites and nail disease. Her baseline disease severity scores included Psoriasis Area Severity Index (PASI), 37.6; Dermatology Life Quality Index (DLQI), 27; Patient Health Questionnaire-9 (PHQ9), 23; Generalized Anxiety Disorder 7-item (GAD7), 17; and tender joint count (TJC), 13.

In light of her deteriorating status and absence of clinical improvement with etanercept biosimilar alone for 9 weeks, she received two intravenous infusions of allogeneic UC-MSCs, 2.57 × 10^6^ cells/kg/infusion (weight 89 kg; total cells per infusion: 229 × 10^6^) 1 week (W) apart, as an adjuvant therapy—a salvage intervention rather than part of a combination therapy planned upfront. Post-MSC infusions, her laboratory and vital signs remained stable. No serious adverse events occurred, although mild cannulation-site thrombophlebitis resolved with conservative management.

By W2, the PASI had reduced to 23 (PASI39), the DLQI had reduced to 10 (−17), with improvements in itch, TJC (−4), PHQ9 (−14), and GAD7 (−12). At W10, the PASI was at its lowest at 21.8 (PASI42). At W24, the PASI had increased to 30.7 despite the continuation of etanercept. She switched to a new biologic—bimekizumab—achieving an absolute PASI8 at W62 (PASI79 from baseline).

### Patient 2

**Figure 2:**
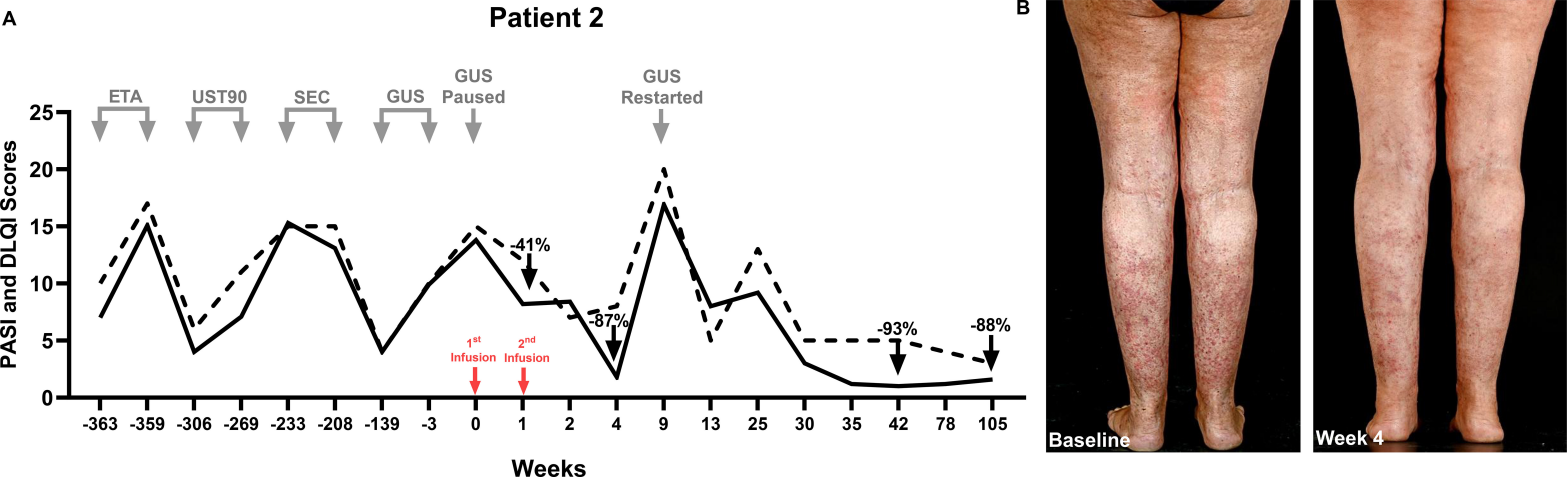
**(A)** Therapeutic timeline of Patient 2. Four biologics across four classes failed in Patient 2. UC-MSCs were administered as a monotherapy after 8 weeks of washout from guselkumab. **(B)** Patient 2’s clinical images at baseline and Week 4, when she experienced an 87% reduction in the PASI. This efficacy diminished by Week 9 when guselkumab, the last failed biologic, was reinitiated at a loading dose. This improved the PASI by 94% from the time of biologic reinitiation, which was sustained through Week 42, and the PASI remained <2 for over 2 years.

A 45-year-old South Asian woman with small-plaque and follicular psoriasis for 25 years presented with breakthrough flares of psoriasis at W5 on guselkumab 2.5 years into treatment (**Figure 2**). She had secondary failure to four biologics across three classes: adalimumab and etanercept biosimilar; ustekinumab (anti-IL-23p40); and secukinumab. Her baseline PASI was 13.8, her DLQI was 15, her PHQ9 was 9, and her GAD7 was 3. She received UC-MSCs as monotherapy, 3.00 × 10^6^ cells/kg/infusion (weight 76.3 kg; total cells per infusion: 229 × 10^6^) 1 week (W) apart, after an 8-week guselkumab washout period. Mild, transient nausea and tachycardia (112 bpm) occurred during infusion, which resolved spontaneously. Laboratory parameters remained stable post-MSCs.

By Day 3, itching was reduced by 90%. At W1, the PASI had decreased to 8.2 (PASI41) and the DLQI had decreased to 12 (−3), and by W4, the PASI and DLQI had reduced to 1.8 (PASI87) and 8 (−7), respectively. This significant improvement in PASI scores was transient, and psoriasis relapsed at W9 post-MSCs, with an absolute PASI of 16.9 and a DLQI of 20; her last ineffective biologic, guselkumab, was reinitiated at a loading dose. Within 4 weeks, the PASI improved by 50% and the DLQI decreased from 20 to 5; she maintained an absolute PASI <2 for over 2 years—her best response to date.

### Patient 3

**Figure 3:**
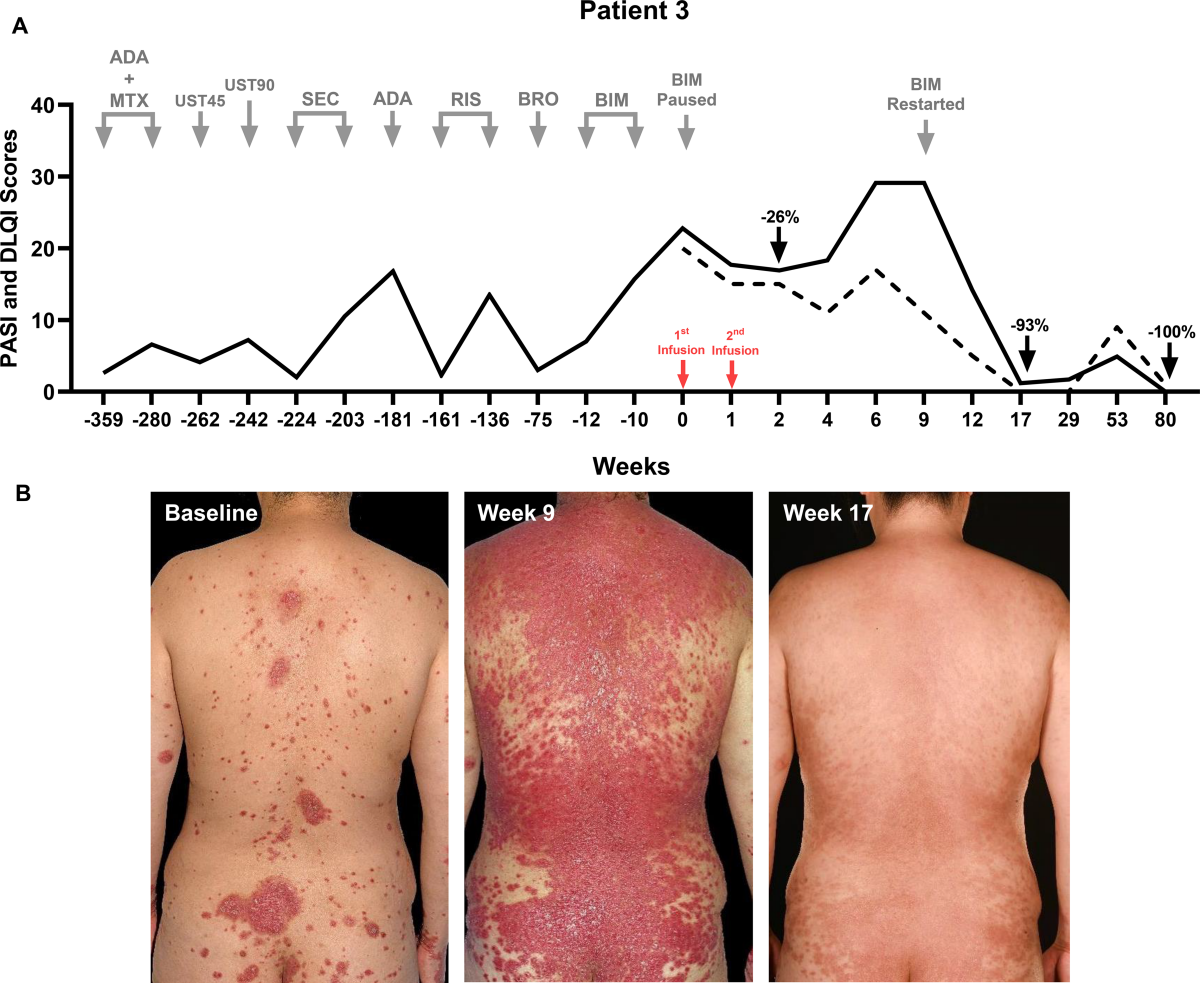
**(A)** Therapeutic timeline of Patient 3. Six biologics across four classes failed in Patient 3. UC-MSCs were administered as a monotherapy after 8 weeks of washout from bimekizumab. **(B)** Patient 3’s clinical images at baseline, at Week 9 when her psoriasis flared post-sunburn, and at Week 17 when she experienced a 93% reduction in the PASI score post-MSCs and a 96% reduction 8 weeks after the reinitiation of a previously failed biologic—bimekizumab. Her absolute PASI remained at 0 at Week 80.

A 47-year-old Chinese woman with long-standing large-plaque psoriasis (**Figure 3**) with vulvar involvement had NAFLD and tested positive for hepatitis B core antibody (she was receiving entecavir treatment). She had secondary failure to five biologics across four classes—adalimumab ± methotrexate, ustekinumab, secukinumab, risankizumab, and brodalumab—with breakthrough flares at W3–4 on bimekizumab (anti-IL-17A/F). Her baseline PASI score was 22.8, with a DLQI of 20.

She received UC-MSCs as monotherapy, 1.96 × 10^6^ cells/kg/infusion (weight 77 kg; total cells per infusion: 151 × 10^6^) 1 week (W) apart, after an 8-week bimekizumab washout period. Transient infusion headaches resolved spontaneously. Vital signs and laboratory parameters remained stable post-treatment. There was no direct benefit from the UC-MSCs (the PASI had reduced to 16.9 and the DLQI had reduced to 5 at W2), followed by a flare (the absolute PASI was 29.1, and the DLQI was 17) post-sunburn during a holiday in Spain. Bimekizumab was restarted at W10, leading to an absolute PASI of 1.2 at W18 and 0 at W80 post-MSC (W70 post-bimekizumab reinitiation).

We performed a biomarker assay that predicts clinical response to MSCs for the three patients with psoriasis, which was previously validated in patients with graft-versus-host disease and Crohn disease (15, 16). This assay is based on the observation that MSCs, upon intravenous infusion, undergo apoptosis induced by recipient cytotoxic cells, and measures the molecular and functional profiles of recipient monocytes exposed to apoptotic MSCs (ApoMSCs). Patient 1 exhibited a borderline positive cytotoxic killing capacity at 11% ApoMSCs; Patient 2 was highly positive at 30%. Patient 3 was intermediate at 18% (**Supplementary Figure 4**).

**Figure 4:**
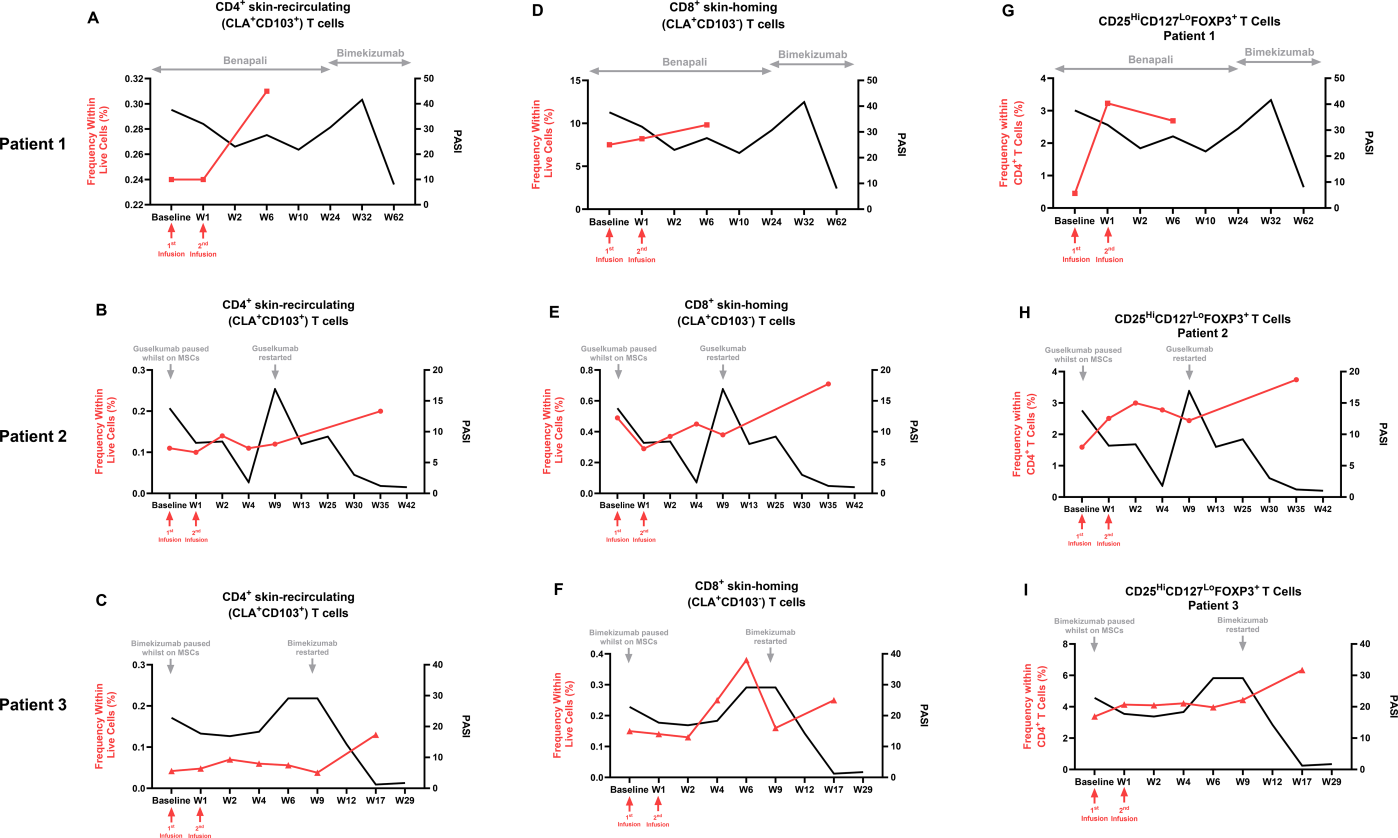
Multi-parameter flow cytometry of peripheral blood mononuclear cells from the three patients before and after UC-MSC therapy and reinitiation of previously failed biologics. Overall increases in the frequency of **(A–C)** CD4^+^CLA^+^CD103^+^ skin-recirculating T cells; **(D–F)** CD8^+^CLA^+^CD103^−^ skin-homing T cells in the peripheral blood of the three patients with psoriasis before and after UC-MSC therapy, both expressed as a percentage within live cells (red line); and **(G-I)** CD25^Hi^CD127^Lo^FoxP3^+^ regulatory T cells, expressed as a percentage of the parent CD4^+^ population, superimposed with the PASI score (black line), before and after UC-MSC infusions in Patient 1 **(A, D, G)**, Patient 2 **(B, E, H)**, and Patient 3 **(C, F, I)**. Red arrows indicate the time of UC-MSC infusions. Gray arrows denote the duration of the biologic/biosimilar or when it was paused or reinitiated. W, Week.

Multi-parameter flow cytometry of all three patients revealed distinct immune profiles at baseline. Specifically, Patients 1 and 3, who had large-plaque psoriasis, had higher frequencies of IL - 17A-producing CD4^+^ and CD8^+^ T cells compared to Patient 2, who had small-plaque, follicular psoriasis and had a higher frequency of interferon (IFN)-γ-producing counterparts (**Supplementary Figure 6**). Interestingly, this observation was also reflected in IL-17A- and IL-22-producing skin recirculating CD4+ and CD8+ T cells, with a similar pattern observed in B cells and type 1 conventional dendritic cells (cDC1). Some patients showed similar trends in some cell subsets of the lymphoid (**Figure 4**, **Supplementary Figure 5**) and myeloid compartments (**Supplementary Figure 7**) following UC-MSC therapy: increased frequencies of CD4^+^ and CD8^+^ skin-recirculating (CLA^+^CD103^+^) memory T cells (17, 18) and CD25^Hi^CD127^Lo^FoxP3^+^ regulatory T cells (**Supplementary Figure 8**), but decreased frequencies of intermediate (CD14^+^CD16^+^) monocytes, associated with clinical improvements. Variation between individuals was noted, and the observations above are based on descriptive rather than statistical analysis due to the small sample size.

## Discussion

MSCs have emerged as a promising therapeutic avenue for patients with severe plaque psoriasis,; however, their role in biologic-refractory cases remains largely unexplored (5, 19–21). To our knowledge, this is the first report describing the use of MSCs as rescue therapy in patients with severe, multiple-biologic-resistant psoriasis, demonstrating an acceptable safety profile and suggesting potential clinical benefits, including a sustained (>2 years), regained response to a previously failed biologic in three selected cases.

Our data suggest that UC-MSCs are well tolerated and safe for at least the duration of follow-up (>2 years), which aligns with previous reports, albeit with a shorter follow-up period (48 weeks). Across four clinical trials administering MSCs to 35 patients with psoriasis, no serious adverse events were reported (19, 20, 22, 23). Similarly, a systematic review concluded that MSC-based therapies are generally safe and may offer clinical benefits in psoriasis management (13, 24). Of interest, Patient 1 had a flare of psoriasis 27–31 weeks after the cessation of UC-MSC therapy, which could reflect disease rebound; however, the underlying mechanism remains unclear and warrants further investigation.

Regarding efficacy, observations from the three patients indicate that UC-MSCs provide clinical benefits, even in cases of severe and biologic-refractory psoriasis. Indeed, the contrast in treatment durations of previous biologics (years) vs. UC-MSCs (1 week) (**Figures 1**-**3**) highlights both the chronicity of prior biologic exposure and the need for new approaches in refractory cases. Previous clinical trials using UC-MSCs reported that up to 75% of patients achieved PASI75 at 24 – 48 weeks (20, 23), albeit with more intensive dosing schedules than ours. Additionally, MSCs from other sources (e.g., bone marrow) have shown efficacy in atopic dermatitis at lower doses (25). Indeed, one of the key challenges with MSC therapy remains the lack of standardized dosing (13), which requires further investigation through larger randomized controlled clinical trials.

An intriguing aspect of our findings is the enhanced responsiveness to previously ineffective or failed biologic therapies following UC-MSC treatment in Patients 2 and 3. While reloading with guselkumab and bimekizumab in Patients 2 and 3, respectively, may have contributed to the observed efficacy, the extent and durability of the response, particularly in the context of prior secondary failure, suggest the possibility that UC-MSC therapy may have also contributed to restoring responsiveness to biologics. This observation may be attributed to the ability of MSCs to reset the immune response, reducing chronic inflammation and restoring sensitivity to biologic agents (26, 27). By modulating the immune milieu, MSCs may diminish the pathogenic T-cell populations and cytokine levels that contribute to biologic resistance (7, 13, 24). Consequently, reintroducing biologics in a less inflammatory environment could enhance their efficacy, as observed in our patients.

A relevant finding related to this intriguing observation of a renewed response to a previously failed biologic treatment is the IMMhance study (28). In this phase 3 randomized trial, the majority of patients with psoriasis who relapsed after withdrawal of risankizumab regained a response upon re-treatment, particularly when reloading doses were applied. However, a key distinction between the IMMhance study and our patients is that IMMhance evaluated planned withdrawal at W28 and re-treatment in patients who responded to risankizumab, while our patients experienced secondary biologic failure. Indeed, secondary biologic failure is rarely reversible by reintroduction of the same failed biologic. Furthermore, the timing and magnitude of the response observed after UC-MSC therapy, particularly the sustained benefit (absolute PASI <2) lasting over 2 years following biologic reinitiation, suggest that a broader immunological reset may have occurred. Given the emerging evidence of MSCs enhancing immune tolerance and reducing inflammatory load (7, 13, 24), we propose that UC-MSC therapy may have created a more permissive immunological environment, enabling guselkumab to regain efficacy.

At a cellular level, deep immunophenotyping revealed distinct baseline immune profiles corresponding to clinical phenotypes, with Patients 1 and 3 displaying a Th17-predominant profile (elevated IL - 17A-producing CD4^+^ and CD8^+^ T cells), which is associated with large-plaque psoriasis, while Patient 2 exhibited a T1-predominant profile (increased IFN-γ-producing T cells), which is linked to her small-plaque, follicular phenotype. Similarly, differences in other immune cell populations were also observed among the three patients (**Supplementary Table 4**): higher frequencies of IL - 17A- and IL - 22-producing skin-recirculating CD4^+^ and CD8^+^ T cells, B cells, and cDC1 were noted in Patients 1 and 3 compared to Patient 2; higher frequencies of IFN-γ-producing CD4^+^ T cells and Mucosal-Associated Invariant T (MAIT) cells, along with IFN-γ-producing skin-recirculating and skin-homing CD4^+^ and CD8^+^ T cells were found in Patient 2 compared to Patients 1 and 3. These differences may explain the varying responses observed with UC-MSC therapy. We also observed that the best clinical response to MSCs correlated with the highest host cytotoxic killing capacity, with Patient 2 (30% ApoMSCs) showing the most dramatic improvement out of the three patients, indicating its potential as a predictive biomarker for MSC responsiveness.

Additionally, we noted that UC-MSCs modulated lymphoid and myeloid cell populations in peripheral blood, which closely coincided with clinical improvement. Our findings indicate that UC-MSC treatment, either alone or as an adjunct to biologic therapy, may inhibit the migration of skin-homing T cells into involved skin in psoriasis, while facilitating the egress of CLA^+^CD103^+^ cells (17, 18) into the peripheral circulation. This observation is consistent with prior findings showing a significant decrease in CD103^+^ T cells in psoriasis-involved skin following treatment with IL - 17A or IL - 23p19 inhibitors (29, 30), suggesting that MSCs may exert similar effects on T-cell migration dynamics (13, 31, 32). Furthermore, phenotypic shifts in monocyte subsets were observed with a reduction in the frequency of CD14^+^CD16^+^ intermediate monocytes correlating with decreased PASI values across all patients. Given that lower frequencies of intermediate monocytes are associated with milder disease in patients with psoriasis (33), this supports the notion that non-classical monocytes exhibit anti-inflammatory properties (34), reinforcing a reduction in inflammatory status.

The immunomodulatory properties of MSCs are probably central to their therapeutic effects in psoriasis. MSCs can modulate both innate and adaptive immune responses by secreting anti-inflammatory cytokines and promoting the development of regulatory immune cells (13, 30, 31). For instance, MSCs have been shown to inhibit T helper (Th) 17 cell proliferation and reduce the production of pro-inflammatory cytokines such as IL - 17 and TNF-α, which play a pivotal role in psoriasis pathogenesis (35–38). Additionally, MSCs can enhance Treg function, restoring immune homeostasis (25–27). In our study, we observed an increase in Treg frequencies following UC-MSC therapy, correlating with clinical improvement. This aligns with previous findings indicating that MSC-induced Treg expansion contributes to therapeutic efficacy in inflammatory diseases (13, 14, 20, 22, 23). Given the role of Tregs in maintaining immune homeostasis and controlling inflammation, modulating their frequency and function may be a key mechanism by which MSCs can reset the recipient’s immune response, enhancing outcomes with previously failed biologics (25–27, 32). Similar studies of MSCs in psoriasis have reported increased frequencies of Tregs and CD4^+^ and CD8^+^ central memory T cells alongside a decrease in circulating Th17 and CD4^+^ naïve T cells. Furthermore, co-culturing of psoriasis patient PBMCs with UC-MSCs significantly inhibited inflammatory cytokine production (IFN-γ, TNF, and IL - 17A) compared to controls (23), reinforcing the immunomodulatory properties of MSCs.

In addition to direct immunomodulation, MSCs may also influence the skin microenvironment by affecting keratinocyte behavior and angiogenesis (33, 34, 39, 40). MSCs secrete anti-inflammatory cytokines and perhaps other factors that inhibit keratinocyte proliferation and promote differentiation, thereby normalizing the aberrant epidermal turnover characteristic of psoriasis plaques (13). Moreover, MSC-derived exosomes have been shown to alleviate psoriasis-like skin inflammation in a mouse model by modulating the IL - 23/IL-17 axis and inhibiting dendritic cell activation, promoting the resolution of psoriasis (21, 37). In an imiquimod-induced psoriasiform model, human UC-MSC-derived exosomes significantly reduced epidermal proliferation, PASI, and the expression of IL - 17, IL - 23, and CCL20 while inhibiting STAT3 phosphorylation (39).

In addition to regulating the skin microenvironment, MSCs are known to inhibit neutrophil activity and plasmacytoid dendritic cell (pDC)-driven type I IFN responses, disrupting feedback loops that perpetuate inflammation in psoriasis (11). This is mediated in part by MSC-derived soluble factors such as TGF-β, IL - 10, PGE2, indoleamine 2,3-dioxygenase (IDO), and tumor necrosis factor-stimulated gene-6 (TSG - 6), along with extracellular vesicles (exosomes) that exert systemic and local anti-inflammatory effects (40–42). In imiquimod mouse models treated with UC-MSCs primed by IFN-γ and TNF-α, TSG - 6 was shown to inhibit neutrophil recruitment by decreasing the expression of CXCL1 with a subsequent reduced level of STAT1 phosphorylation in keratinocytes (40–42).

Collectively, our findings support the view that UC-MSCs exert their therapeutic effect on psoriasis via layered immunological and tissue-level mechanisms: suppression of pathogenic T-cell subsets; expansion of Tregs; modulation of cytokine and chemokine networks; inhibition of dendritic cell activity; and regulation of monocyte subsets, keratinocyte behavior, and the overall inflammatory burden. Our case series contributes to this growing body of evidence and highlights the need for controlled studies to further define these mechanisms and identify predictive biomarkers of response.

Limitations of this case series include the small sample size that limited statistical analysis in immunological profiling data, variations in previous treatments, timelines of clinical assessments and immuno-phenotyping, and potential confounders such as short courses of topical corticosteroids during psoriasis flare-ups post-MSC therapy. In Patient 1, the concurrent use of an etanercept biosimilar along with UC-MSCs represents a further confounding factor in the interpretation of the clinical response. Moreover, the duration of UC-MSC therapy was limited owing to the constraints of compassionate-use access, which provided only 2–3 × 10^6^ cells/kg, two infusions per patient, 1 week apart. Nonetheless, the depth of immune profiling and long-term follow-up strengthen our findings.

In conclusion, our study contributes to the growing body of evidence supporting the safety and potential efficacy of MSC therapy for severe, and in particular, biologic-refractory psoriasis. The observed clinical improvements and novel durable enhanced responsiveness to previously failed biologics underscore the promise of MSCs as a therapeutic strategy. Undoubtedly, several challenges remain, including the optimal tissue source (43), dosing regimen (44), mechanisms of action (44–46), predictive biomarkers of treatment response, and long-term safety and efficacy. Further investigations of randomized controlled trials with larger sample sizes, perhaps in patients with psoriasis at an earlier stage in their therapeutic journey, are warranted to elucidate the mechanisms of action and therapeutic potential of MSCs in the management of psoriasis.

## Data Availability

The datasets presented in this study can be found in online repositories. The names of the repository/repositories and accession number(s) can be found in the article/**Supplementary Material**.
