# Supplementary material for: Mesenchymal stromal cells as rescue therapy in biologic-refractory psoriasis: insights from a case series
Source: Front Immunol. 2025 Sep 3;16:1656724. doi: 10.3389/fimmu.2025.1656724 (PMC12440939; doi:10.3389/fimmu.2025.1656724)
Supplement: Supplementary file 1 [file DataSheet1.docx]

**SUPPLEMENTARY MATERIALS**

**Patient consent, monitoring and sample collection**

Three adult females, aged 44, 46 and 47 years from the psoriasis clinic at the Department of Dermatology, King’s College Hospital (KCH), London, UK were consented for treatment with allogeneic UC-MSCs on compassionate grounds between 2022 and 2023. These three patients were carefully chosen based on clinical grounds: severe psoriasis PASI≥12 with high DLQI >10; and had failed multiple lines of biologics. The protocol was approved by the KCH Biological Safety Committee and New Clinical Procedures Committee, which permitted the use of MSCs on compassionate grounds.

Patients were examined and vital signs monitored before and at least an hour after treatment. Pre-MSC blood tests included full blood count (FBC), renal (U&E) and liver (LFT) profiles, ESR, CRP, HIV, HTLV1 and 2, Syphilis, Hepatitis (Hep) B surface antigen, HepB surface and core antibodies and HepC IgG. Patients were reviewed by telephone 24 hours and 3 days after the individual infusions of MSCs and in person at W2, every 3 months and if necessary if psoriasis flared.

From the three patients, informed consent was obtained and the use of patient blood samples was approved by the appropriate ethics committee (REC Reference: 11/HO802/7; IRAS Number: 71048).

**Healthy volunteers**

The HVs were recruited at St John’s Institute of Dermatology, and peripheral blood mononuclear cells (PBMCs) from 10 individuals among this group were used in the study (**Supplementary Table 1**). All healthy volunteers provided informed consent and the use of PBMCs was approved by the ethics committee (REC Reference: 06/Q0704/18).

**Supplementary Table 1. Demographics of healthy volunteers.**

**
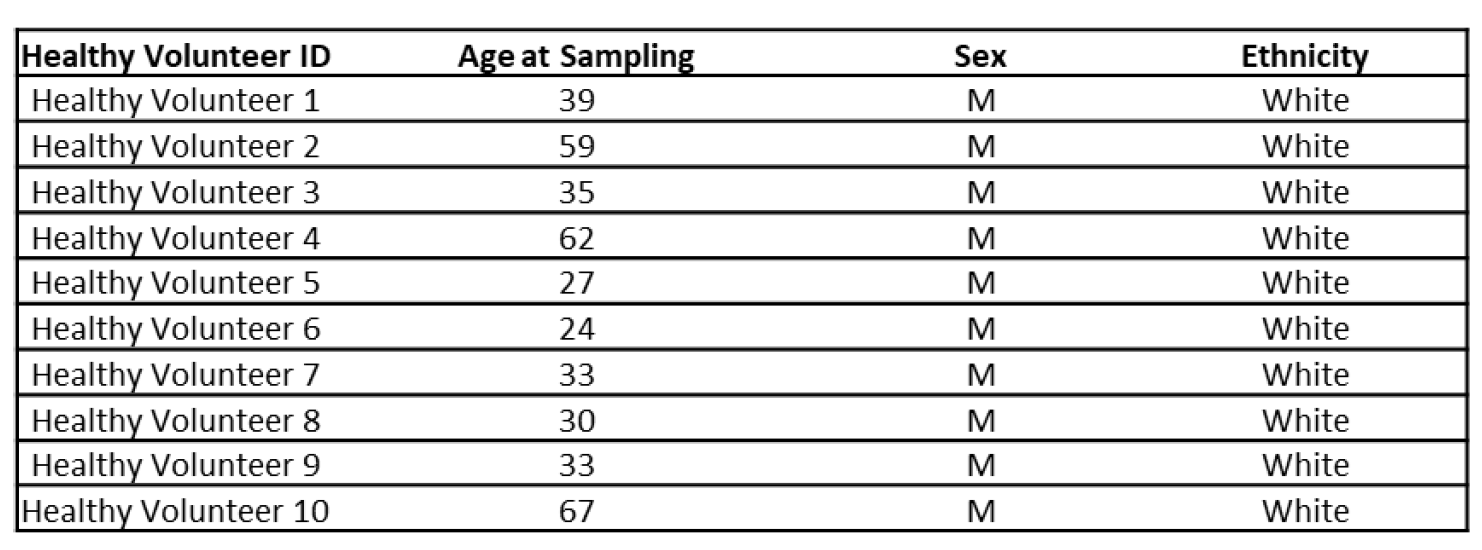
**

**Procedure**

After standard immunomodulatory therapy screening, two intravenous infusions of allogeneic UC-MSCs were administered at 1.5–3x10^6^ cells/kg a week (W) apart. Intravenous chlorphenamine premedication was administered prior to UC-MSCs. P1, owing to psoriasis severity, received UC-MSCs as adjuvant therapy alongside etanercept until W24 when it was switched to a new biologic, bimekizumab. P2 and P3 received UC-MSCs as monotherapy after stopping biologics, guselkumab and bimekizumab respectively, for >8 weeks as washout. Upon psoriasis relapse at W9, the previously failed biologics were re-initiated at standard loading doses.

**PBMC isolation and cryopreservation**

PBMCs were isolated and cryopreserved as previously described by Andres-Ejarque et al., 2021.^1^ Briefly, blood samples were collected at KCH, in BD Vacutainer™ Hemogard™ Closure Plastic K2-Edta Tube (BD Biosciences). PBMCs were isolated in Leucosep tubes (Greiner Bio-One) using Ficoll-Paque PLUS (Sigma-Aldrich) and density gradient centrifugation. PBMCs were then resuspended in RPMI-1640 (Gibco™) comprising 11.25% Human Serum Albumin (Gemini Bio-Products) and 10% dimethyl sulfoxide (Sigma-Aldrich) and cryopreserved in liquid nitrogen until further use.

**Biomarker assays**

Apoptotic assay: MSCs were stained with 2.5$\mu M$ CellTraceTM Violet (Thermo Fisher Scientific, UK) and plated overnight (5x10^4^ cells/well) in a 24-well plate. The day after, PBMCs were freshly isolated from psoriasis patients’ blood samples and added to the MSC monolayer at a PBMC:MSC ratio of 20:1. MSC alone were used as controls. After 4 hours, supernatant was removed and cells were collected for flow cytometry analysis. Apoptosis was measured by using Annexin-V apoptotsis detection kit (BD Bioscencies). MSCs were identified as Violet+ cells. The percentage of Annexin-V^+^7-AAD^-^ MSC population was evaluated and referred to as ApoMSCs.

ELISA assay: Cell supernatants were collected from MSCs alone and MSC:PBMC co-cultures after 24 hours and levels of PGE2 were quantified by ELISA (Caymann, USA) according to the manufacturer’s instructions.

**PBMCs phenotyping**

Cryopreserved PBMCs were thawed, and the cell count, and viability recorded using a NucleoCounter NC-200 (Chemometec). PBMCs samples with a cell count >5 million and a viability >85% were resuspended in RPMI-1640 medium supplemented with 10% heat-inactivated fetal calf serum (FCS; Gibco™) and 1% Penicillin-Streptomycin at a concentration of 1.5 million cells per mL. 2 million PBMCs were centrifuged and stained with LIVE/DEAD™ fixable blue stain (Invitrogen) for 15 minutes at room temperature. Cells were then washed in FACS buffer comprising phosphate-buffered saline and 2% bovine serum albumin and stained with antibodies from Panel A or Panel C (**Supplementary Table 2**), for 30 minutes at 4°C. Cells were then fixed with Human FoxP3 Buffer A (BD Biosciences) according to the manufacturer’s instructions. The remaining PBMCs were rested overnight in an incubator at 37°C and 5% CO_2_.

On the next day, the cell count and viability were recorded for the remaining cells rested overnight. Samples with a cell count more than 3 million and a viability greater than 85% were equally split into two 5mL FACS tubes (VWR) labelled “unstimulated” and “stimulated”. Both tubes were stained with LIVE/DEAD™ fixable blue stain, after which were washed with FACS buffer and stained with surface antibodies from Panel B (**Supplementary Table 2**) for 30 minutes at 4°C. Cells were then fixed with Human FoxP3 Buffer A and permeabilized with FoxP3 Buffer B (BD Biosciences) according to the manufacturer’s instructions. Finally, cells were stained with intracellular antibodies from Panel B (**Supplementary Table 2**) for 60 minutes at 4°C, washed, and resuspended with FACS buffer until acquisition on the flow cytometer.

**Supplementary Table 2. Antibodies used in the flow cytometry panel.**

| **Panel** | **Marker** | **Fluorochrome** | **Clone** | **Manufacturer** | **Catalogue Number** |
| --- | --- | --- | --- | --- | --- |
| **Panel A** | CD3 | BV786 | SK7 | BD Biosciences | 563800 |
|  | CD56 | PE-CF594 | NCAM16.2 |  | 564849 |
|  | CD20 | BUV395 | 2H7 |  | 563782 |
|  | CD14 | BV510 | MφP9 |  | 563079 |
|  | CD16 | APC | 3G8 |  | 561249 |
|  | HLA-DR | APC-R700 | G46-6 |  | 565127 |
|  | CD123 | BV421 | 7G3 |  | 563362 |
|  | CD11c | PE-Cy7 | B-Ly6 |  | 561356 |
|  | CD141 | BB515 | 1A4 |  | 565084 |
|  | CD1c | PerCPCy5.5 | F10/21A3 |  | 565423 |
|  | Fixable L/D | DAPI |  | Invitrogen | L34961 |
| **Panel B** | CD3 | BUV395 | SK7 | BD Biosciences | 564001 |
|  | CD4 | APC-Cy7 | SK3 |  | 641389 |
|  | CD8 | BV510 | RPA-T8 |  | 563256 |
|  | CD161 | BV421 | DX12 |  | 562615 |
|  | CD103 | BV650 | Ber-ACT8 |  | 743653 |
|  | CLA | BV605 | HECA-452 |  | 563960 |
|  | CCR6 | PE-Cy7 | 11A9 |  | 560620 |
|  | CD56 | BV786 | NCAM16.2 |  | 564058 |
|  | IL-17A | PE | N49-653 |  | 560487 |
|  | IFNγ | FITC | 4S.B3 |  | 561057 |
|  | Granzyme B | AF700 | GB11 |  | 560213 |
|  | TCRVa7.2 | PE-CF594 | 3C10 | BioLegend | 351730 |
|  | IL-22 | APC | IL22JOP | eBioscience | 17-7222-80 |
|  | Fixable L/D | DAPI |  | Invitrogen | L34961 |
| **Panel C** | CD3 | AF700 | UCHT1 | BD Biosciences | 557943 |
|  | CD4 | BUV395 | SK3 |  | 563550 |
|  | CD25 | CF594 | M-A251 |  | 562403 |
|  | CD127 | BV786 | HIL-7R-M21 |  | 563324 |
|  | CD45RA | BUV496 | HI100 |  | 750258 |
|  | OX-40 | PC5 | ACT35 |  | 551500 |
|  | CD161 | BV650 | DX12 |  | 563864 |
|  | GATA3 | PerCP5.5 | 16E10A23 |  | 653812 |
|  | HELIOS | APC | 22F6 |  | 137222 |
|  | RORγt | AF488 | Q21-559 |  | 563621 |
|  | CD39 | BV510 | A1 |  | 567526 |
|  | PD1 | BV711 | EH12.2H7 | BioLegend | 329928 |
|  | CD27 | BV605 | O323 |  | 302830 |
|  | CTLA4 | BV421 | BNI3 |  | 369606 |
|  | FOXP3 | PE | PCH101 | ThermoFisher | 12-4776-42 |
|  | Fixable Viability Stain | 780 |  | BD Biosciences | 565388 |

**Data acquisition by flow cytometry**

Samples stained with antibodies from Panels A, B and C (**Supplementary Table 2**) were acquired on the BD LSRFortessa™ Cell Analyzer using BD Standardized Application Setup. This allows the same laser settings to be used across different experiments, thereby minimizing batch effects and enabling the comparison of cell frequency and cytokine production between samples run in different batches. 250,000 live cells were recorded using the BD FACSDiva software and .fcs files for each sample were generated.

**Manual Gating**

Immune cells of interest were identified through manual gating using FlowJo™ v10.8. The frequency of manually identified macro-populations was represented as % within live cells, while cytokine-producing cells were represented as % within their parent population.

**Statistical analysis**

Due to sample size limitations, we used descriptive statistics to explain the changes in cell frequency observed during MSC treatment. Statistical comparisons tests between healthy volunteers and the MSC-treated patient cohort were run on GraphPad Prism v10.0. D'Agostino Pearson Normality tests were run to assess distribution of data. Mann-Whitney Tests with follow-up Benjamini–Hochberg approach (False Discovery Rate, FDR, with a q value of 0.05) were used to control for multiple comparisons.

**Supplementary Table 3.** Table illustrating the difference in frequency of immune lymphoid cell subsets expressed either as a percentage of the parent macro-population or within live cells, in healthy volunteers (n=8) and psoriasis patients (n=3) at baseline. Mann-Whitney Test and FDR multiple comparisons test. *p<0.05, ns – non-significant. FDR q<0.05.

| **Cell Subset** | **Frequency (%)** | | **Test** | **P Value** | **FDR corrected q value** |
| --- | --- | --- | --- | --- | --- |
|  | **Healthy Volunteers**  **(n=8)** | **MSC-Ps**  **(n=3)** |  |  |  |
| CD4^+^ T cells | 41.50 | 39.10 | Mann Whitney Test | 0.9371 | 0.9731 |
| CD4^+^IL-17A^+^ T cells | 1.06 | 1.02 | Mann Whitney Test | 0.6678 | 0.8899 |
| CD4^+^IL-22^+^ T cells | 0.52 | 0.49 | Mann Whitney Test | 0.7867 | 0.8899 |
| CD4^+^IFNγ^+^ T cells | 10.00 | 22.80 | Mann Whitney Test | 0.1455 | 0.4074 |
| CD4^+^CLA^+^CD103^+^ T cells | 0.42 | 0.11 | Mann Whitney Test | **0.0485 (*)** | 0.3430 |
| CD4^+^CLA^+^CD103^+^IL-17A^+^ T cells | 1.10 | 9.89 | Mann Whitney Test | 0.0769 | 0.3418 |
| CD4^+^CLA^+^CD103^+^IL-22^+^ T cells | 7.85 | 14.4 | Mann Whitney Test | 0.1119 | 0.3418 |
| CD4^+^CLA^+^CD103^+^IFNγ^+^ T cells | 3.62 | 5.49 | Mann Whitney Test | **0.0140 (*)** | 0.1960 |
| CD4^+^CLA^+^CD103^-^ T cells | 12.05 | 4.78 | Mann Whitney Test | 0.0769 | 0.3481 |
| CD4^+^CLA^+^CD103^-^IL-17A^+^ T cells | 2.34 | 1.63 | Mann Whitney Test | 0.8112 | 0.8899 |
| CD4^+^CLA^+^CD103^-^IL-22^+^ T cells | 1.37 | 1.67 | Mann Whitney Test | 0.5734 | 0.8899 |
| CD4^+^CLA^+^CD103^-^IFNγ^+^ T cells | 8.80 | 9.89 | Mann Whitney Test | 0.2867 | 0.6689 |
| CD8^+^ T cells | 24.05 | 19.50 | Mann Whitney Test | 0.8357 | 0.8899 |
| CD8^+^IL-17A^+^ T cells | 0.67 | 0.48 | Mann Whitney Test | 0.1713 | 0.4360 |
| CD8^+^IL-22^+^ T cells | 0.39 | 0.35 | Mann Whitney Test | 0.7168 | 0.8899 |
| CD8^+^IFNγ^+^ T cells | 45.00 | 56.20 | Mann Whitney Test | 0.4685 | 0.8198 |
| CD8^+^CLA^+^CD103^+^ T cells | 0.48 | 0.05 | Mann Whitney Test | 0.0121 | 0.1960 |
| CD8^+^CLA^+^CD103^+^IL-17A^+^ T cells | 4.07 | 9.50 | Mann Whitney Test | 0.1119 | 0.3481 |
| CD8^+^CLA^+^CD103^+^IL-22^+^ T cells | 4.29 | 6.50 | Mann Whitney Test | 0.8112 | 0.8899 |
| CD8^+^CLA^+^CD103^+^IFNγ^+^ T cells | 49.20 | 17.50 | Mann Whitney Test | 0.049 | 0.3430 |
| CD8^+^CLA^+^CD103^-^ T cells | 4.09 | 0.49 | Mann Whitney Test | 0.3706 | 0.7412 |
| CD8^+^CLA^+^CD103^-^IL-17A^+^ T cells | 0.79 | 0.68 | Mann Whitney Test | 0.6923 | 0.8899 |
| CD8^+^CLA^+^CD103^-^IL-22^+^ T cells | 0.56 | 0.59 | Mann Whitney Test | 0.6853 | 0.8899 |
| CD8^+^CLA^+^CD103^-^IFNγ^+^ T cells | 34.50 | 23.40 | Mann Whitney Test | 0.3706 | 0.7412 |
| CD8^+^ MAIT cells | 4.23 | 0.28 | Mann Whitney Test | 0.1119 | 0.3413 |
| CD8^+^IL-17A^+^ MAIT cells | 1.77 | 2.06 | Mann Whitney Test | 0.4685 | 0.8198 |
| CD8^+^IL-22^+^ MAIT cells | 0.86 | 0.66 | Mann Whitney Test | 0.9727 | 0.9731 |
| CD8^+^IFNγ^+^ MAIT cells | 49.20 | 30.00 | Mann Whitney Test | 0.6923 | 0.8899 |
| Tregs (CD25^Hi^CD127^Lo^FOXP3^+^ T Cells) | 5.69 | 1.59 | Mann Whitney Test | 0,1000 | 0.2222 |

**Changes in the immune profiles after UC-MSCs which corresponded with clinical status**

Next, we detected changes in the frequency of lymphoid and myeloid cell compartments during (W1) and after UC-MSCs in all patients (**Supplementary** **Table 4**), including skin-homing and skin-recirculating memory T cells, regulatory T cells (Tregs) and non-classical monocytes.

**Supplementary Table 4**. Table illustrating the frequency of immune lymphoid and myeloid cell subsets expressed either as a percentage of the parent macro-population or within live cells, in psoriasis patients, before and after treatment with UC-MSCs over 35 weeks (n=3).

| **Cell Subset** | **Patient** | **Frequency (%)** | | | | | | | |
| --- | --- | --- | --- | --- | --- | --- | --- | --- | --- |
|  |  | **Baseline** | **W1** | **W2** | **W4** | **W6** | **W9** | **W17** | **W35** |
| CD4^+^ T cells | 1 | 29.9 | 29.2 |  |  | 26.6 |  |  |  |
|  | 2 | 57.3 | 54.6 | 57.1 | 50.5 |  | 50.9 |  | 47.3 |
|  | 3 | 39.1 | 42.2 | 44 | 45.2 | 45.5 | 36 | 37.8 |  |
| CD4^+^IL-17A^+^ T cells | 1 | 1.02 | 0.95 |  |  | 0.88 |  |  |  |
|  | 2 | 0.35 | 0.46 | 0.37 | 0.38 |  | 0.39 |  | 0.43 |
|  | 3 | 1.45 | 1.56 | 1.32 | 1.59 | 1.07 | 0.89 | 2.09 |  |
| CD4^+^IL-22^+^ T cells | 1 | 0.49 | 0.40 |  |  | 0.42 |  |  |  |
|  | 2 | 0.44 | 0.61 | 0.48 | 0.61 |  | 0.49 |  | 0.23 |
|  | 3 | 0.59 | 0.67 | 0.61 | 0.70 | 0.47 | 0.36 | 0.88 |  |
| CD4^+^IFNγ^+^ T cells | 1 | 22.8 | 22.7 |  |  | 21.5 |  |  |  |
|  | 2 | 30.6 | 39.6 | 27.7 | 34.4 |  | 32 |  | 30 |
|  | 3 | 12.4 | 14.1 | 11.6 | 16.6 | 10.4 | 14.1 | 11.1 |  |
| CD4^+^CLA^+^CD103^+^ T cells | 1 | 0.24 | 0.24 |  |  | 0.31 |  |  |  |
|  | 2 | 0.11 | 0.10 | 0.14 | 0.11 |  | 0.12 |  | 0.20 |
|  | 3 | 0.04 | 0.05 | 0.07 | 0.06 | 0.06 | 0.04 | 0.13 |  |
| CD4^+^CLA^+^CD103^+^IL-17A^+^ T cells | 1 | 9.89 | 4.30 |  |  | 7.69 |  |  |  |
|  | 2 | 3.24 | 2.78 | 1.42 | 4.28 |  | 1.44 |  | 4.52 |
|  | 3 | 11.5 | 9.90 | 6.50 | 10.3 | 8.90 | 5.80 | 5.20 |  |
| CD4^+^CLA^+^CD103^+^IL-22^+^ T cells | 1 | 15.4 | 11.8 |  |  | 15.4 |  |  |  |
|  | 2 | 9.19 | 7.78 | 10.3 | 12.8 |  | 8.13 |  | 13.3 |
|  | 3 | 14.4 | 21.3 | 17.3 | 20.2 | 15.1 | 7.25 | 15.1 |  |
| CD4^+^CLA^+^CD103^+^IFNγ^+^ T cells | 1 | 5.49 | 4.30 |  |  | 0.96 |  |  |  |
|  | 2 | 8.65 | 6.11 | 4.96 | 6.95 |  | 6.22 |  | 7.91 |
|  | 3 | 4.81 | 8.80 | 4.80 | 10.2 | 7.90 | 4.35 | 9.80 |  |
| CD4^+^CLA^+^CD103^-^ T cells | 1 | 4.95 | 4.87 |  |  | 5.14 |  |  |  |
|  | 2 | 4.78 | 3.98 | 4.92 | 4.78 |  | 5.61 |  | 5.93 |
|  | 3 | 3.06 | 2.83 | 4.11 | 4.53 | 4.35 | 3.00 | 4.16 |  |
| CD4^+^CLA^+^CD103^-^IL-17A^+^ T cells | 1 | 1.35 | 1.17 |  |  | 1.02 |  |  |  |
|  | 2 | 1.63 | 1.85 | 1.67 | 2.00 |  | 1.66 |  | 2.51 |
|  | 3 | 5.70 | 6.70 | 4.82 | 2.22 | 3.01 | 1.23 | 8.7 |  |
| CD4^+^CLA^+^CD103^-^IL-22^+^ T cells | 1 | 0.85 | 0.64 |  |  | 0.70 |  |  |  |
|  | 2 | 1.67 | 2.21 | 1.45 | 2.28 |  | 1.39 |  | 2.27 |
|  | 3 | 2.68 | 2.93 | 2.58 | 2.50 | 3.43 | 0.65 | 4.34 |  |
| CD4^+^CLA^+^CD103^-^IFNγ^+^ T cells | 1 | 9.89 | 9.17 |  |  | 7.31 |  |  |  |
|  | 2 | 20.3 | 18.3 | 16.8 | 18.6 |  | 17.3 |  | 18.3 |
|  | 3 | 8.61 | 12.4 | 10.2 | 15.6 | 8.67 | 9.78 | 8.41 |  |
| CD8^+^ T cells | 1 | 45.8 | 45.7 |  |  | 47.7 |  |  |  |
|  | 2 | 19.5 | 14.0 | 13.8 | 16.3 |  | 12.1 |  | 23.5 |
|  | 3 | 18.6 | 18.0 | 17.0 | 17.1 | 18.7 | 18.3 | 16.5 |  |
| CD8^+^IL-17A^+^ T cells | 1 | 0.52 | 0.65 |  |  | 0.48 |  |  |  |
|  | 2 | 0.18 | 0.27 | 0.26 | 0.28 |  | 0.23 |  | 0.36 |
|  | 3 | 0.48 | 0.61 | 0.43 | 0.56 | 0.56 | 0.40 | 0.75 |  |
| CD8^+^IL-22^+^ T cells | 1 | 0.35 | 0.45 |  |  | 0.28 |  |  |  |
|  | 2 | 0.29 | 0.39 | 0.38 | 0.65 |  | 0.25 |  | 0.39 |
|  | 3 | 0.38 | 0.46 | 0.34 | 0.49 | 0.92 | 0.42 | 0.59 |  |
| CD8^+^IFNγ^+^ T cells | 1 | 56.2 | 53.8 |  |  | 46.1 |  |  |  |
|  | 2 | 68.3 | 75.3 | 64.9 | 72.7 |  | 69.0 |  | 66.8 |
|  | 3 | 36.1 | 44.4 | 42.0 | 51.1 | 35.0 | 45.4 | 41.6 |  |
| CD8^+^CLA^+^CD103^+^ T cells | 1 | 0.10 | 0.12 |  |  | 0.12 |  |  |  |
|  | 2 | 0.03 | 0.02 | 0.03 | 0.04 |  | 0.03 |  | 0.09 |
|  | 3 | 0.05 | 0.06 | 0.08 | 0.10 | 0.23 | 0.05 | 0.13 |  |
| CD8^+^CLA^+^CD103^+^IL-17A^+^ T cells | 1 | 9.50 | 6.44 |  |  | 4.53 |  |  |  |
|  | 2 | 4.94 | 4.48 | 9.76 | 6.33 |  | 10.3 |  | 10.5 |
|  | 3 | 11.1 | 15.6 | 14.0 | 11.7 | 8.22 | 10.8 | 11.5 |  |
| CD8^+^CLA^+^CD103^+^IL-22^+^ T cells | 1 | 6.5 | 4.72 |  |  | 6.17 |  |  |  |
|  | 2 | 2.47 | 2.99 | 4.88 | 3.80 |  | 0.01 |  | 4.51 |
|  | 3 | 8.60 | 17.5 | 10.0 | 8.90 | 7.90 | 10.9 | 7.90 |  |
| CD8^+^CLA^+^CD103^+^IFNγ^+^ T cells | 1 | 17.5 | 19.3 |  |  | 20.6 |  |  |  |
|  | 2 | 40.7 | 40.3 | 40.2 | 50.6 |  | 46.6 |  | 33.1 |
|  | 3 | 11.4 | 22.9 | 19.0 | 27.3 | 24.8 | 16.3 | 19.2 |  |
| CD8^+^CLA^+^CD103^-^ T cells | 1 | 7.50 | 8.21 |  |  | 9.82 |  |  |  |
|  | 2 | 0.49 | 0.29 | 0.37 | 0.45 |  | 0.38 |  | 0.71 |
|  | 3 | 0.15 | 0.14 | 0.13 | 0.25 | 0.38 | 0.16 | 0.25 |  |
| CD8^+^CLA^+^CD103^-^IL-17A^+^ T cells | 1 | 0.68 | 0.69 |  |  | 0.53 |  |  |  |
|  | 2 | 0.51 | 0.61 | 1.66 | 1.03 |  | 1.12 |  | 1.56 |
|  | 3 | 1.73 | 1.92 | 2.73 | 1.35 | 1.74 | 2.17 | 1.98 |  |
| CD8^+^CLA^+^CD103^-^IL-22^+^ T cells | 1 | 0.59 | 0.58 |  |  | 0.46 |  |  |  |
|  | 2 | 0.51 | 0.61 | 0.41 | 0.43 |  | 0.01 |  | 0.86 |
|  | 3 | 0.99 | 1.29 | 1.11 | 0.77 | 1.56 | 1.18 | 1.31 |  |
| CD8^+^CLA^+^CD103^-^IFNγ^+^ T cells | 1 | 11.9 | 10.5 |  |  | 7.84 |  |  |  |
|  | 2 | 34.4 | 34.2 | 27.4 | 32.5 |  | 29.7 |  | 32.1 |
|  | 3 | 23.4 | 30.4 | 33.1 | 38.8 | 17.1 | 36.6 | 29.4 |  |
| CD8^+^ MAIT cells | 1 | 0.11 | 0.08 |  |  | 0.09 |  |  |  |
|  | 2 | 0.28 | 0.16 | 0.14 | 0.22 |  | 0.17 |  | 0.25 |
|  | 3 | 1.50 | 1.50 | 1.10 | 1.30 | 1.09 | 1.08 | 0.89 |  |
| CD8^+^IL-17A^+^ MAIT cells | 1 | 14.3 | 15.9 |  |  | 19.8 |  |  |  |
|  | 2 | 2.06 | 1.79 | 4.22 | 1.97 |  | 2.76 |  | 5.29 |
|  | 3 | 1.51 | 2.46 | 2.37 | 2.01 | 3.69 | 2.70 | 2.38 |  |
| CD8^+^IL-22^+^ MAIT cells | 1 | 4.29 | 3.41 |  |  | 2.83 |  |  |  |
|  | 2 | 0.51 | 0.40 | 1.11 | 0.99 |  | 0.18 |  | 1.98 |
|  | 3 | 0.66 | 0.57 | 0.76 | 0.80 | 1.23 | 0.67 | 0.60 |  |
| CD8^+^IFNγ^+^ MAIT cells | 1 | 30.0 | 33.0 |  |  | 21.7 |  |  |  |
|  | 2 | 67.8 | 63.3 | 59.1 | 54.8 |  | 51.7 |  | 54.3 |
|  | 3 | 22.6 | 37.5 | 34.2 | 56.4 | 27.5 | 36.3 | 43.2 |  |
| Tregs (CD25^Hi^CD127^Lo^FOXP3^+^ T Cells) | 1 | 0.45 | 3.23 |  |  | 2.69 |  |  |  |
|  | 2 | 1.59 | 2.51 | 3.00 | 2.78 |  | 2.44 |  | 3.74 |
|  | 3 | 3.38 | 4.14 | 4.09 | 4.22 | 3.96 | 4.43 | 6.34 |  |
| B Cells | 1 | 16.8 | 19.4 |  |  | 19.9 |  |  |  |
|  | 2 | 3.63 | 3.53 | 4.20 | 3.57 |  | 2.98 |  | 2.88 |
|  | 3 | 11.8 | 11.8 | 11.7 | 9.94 | 10.6 | 10.8 | 16.7 |  |
| Monocytes | 1 | 9.46 | 8.57 |  |  | 5.94 |  |  |  |
|  | 2 | 11.0 | 5.29 | 5.25 | 8.54 |  | 12.9 |  | 24.0 |
|  | 3 | 19.2 | 21.3 | 14.5 | 6.30 | 17.0 | 15.0 | 11.8 |  |
| Classical Monocytes (CD14^+^CD16^-^) | 1 | 90.9 | 89.9 |  |  | 85.2 |  |  |  |
|  | 2 | 83.5 | 85.3 | 87.5 | 81.7 |  | 78.9 |  | 90.8 |
|  | 3 | 90.3 | 95.1 | 94.8 | 90.9 | 93.7 | 92.6 | 92.0 |  |
| Intermediate Monocytes (CD14^+^CD16^+^) | 1 | 4.24 | 3.94 |  |  | 3.21 |  |  |  |
|  | 2 | 7.61 | 5.77 | 5.28 | 4.05 |  | 9.90 |  | 4.75 |
|  | 3 | 5.23 | 1.91 | 1.88 | 3.47 | 2.43 | 3.73 | 4.07 |  |
| Non-Classical Monocytes (CD14^Lo^CD16^+^) | 1 | 2.00 | 2.80 |  |  | 5.10 |  |  |  |
|  | 2 | 1.77 | 3.83 | 1.81 | 3.23 |  | 4.18 |  | 1.27 |
|  | 3 | 1.55 | 0.87 | 0.75 | 1.36 | 0.76 | 1.62 | 2.00 |  |
| Myeloid DCs | 1 | 0.33 | 0.39 |  |  | 0.33 |  |  |  |
|  | 2 | 0.84 | 0.40 | 0.53 | 0.77 |  | 0.84 |  | 0.61 |
|  | 3 | 0.60 | 0.31 | 0.44 | 0.44 | 0.29 | 0.40 | 0.82 |  |
| cDC1 Cells (CD1c^+^CD141^+^) | 1 | 6.94 | 11.0 |  |  | 7.92 |  |  |  |
|  | 2 | 10.4 | 9.89 | 8.15 | 7.31 |  | 9.30 |  | 5.80 |
|  | 3 | 6.12 | 6.64 | 5.13 | 3.27 | 5.95 | 4.61 | 4.95 |  |
| cDC2 Cells (CD1c^+^CD141^-^) | 1 | 35.5 | 32.9 |  |  | 42.8 |  |  |  |
|  | 2 | 30.9 | 29.7 | 31.2 | 28.3 |  | 30.3 |  | 40.8 |
|  | 3 | 51.3 | 47.8 | 50.6 | 54.4 | 47.1 | 42.9 | 58.1 |  |
| DN Cells (CD1c^-^CD141^-^) | 1 | 50.9 | 45.7 |  |  | 40.5 |  |  |  |
|  | 2 | 49.4 | 50.6 | 51.9 | 57.0 |  | 51.9 |  | 40.9 |
|  | 3 | 36.4 | 36.0 | 37.2 | 36.1 | 39.6 | 43.9 | 29.0 |  |
| Plasmacytoid DCs | 1 | 0.13 | 0.12 |  |  | 0.12 |  |  |  |
|  | 2 | 0.36 | 0.23 | 0.43 | 0.39 |  | 0.50 |  | 0.61 |
|  | 3 | 0.38 | 0.28 | 0.34 | 0.26 | 0.17 | 0.33 | 0.53 |  |

***Cells highlighted in orange are represented in main Figure 4A-L with numbers in red illustrating the most notable increase in frequency of CD4+ and CD8+ skin-homing and skin-recirculating T cells in peripheral blood of Patients 1-3.***


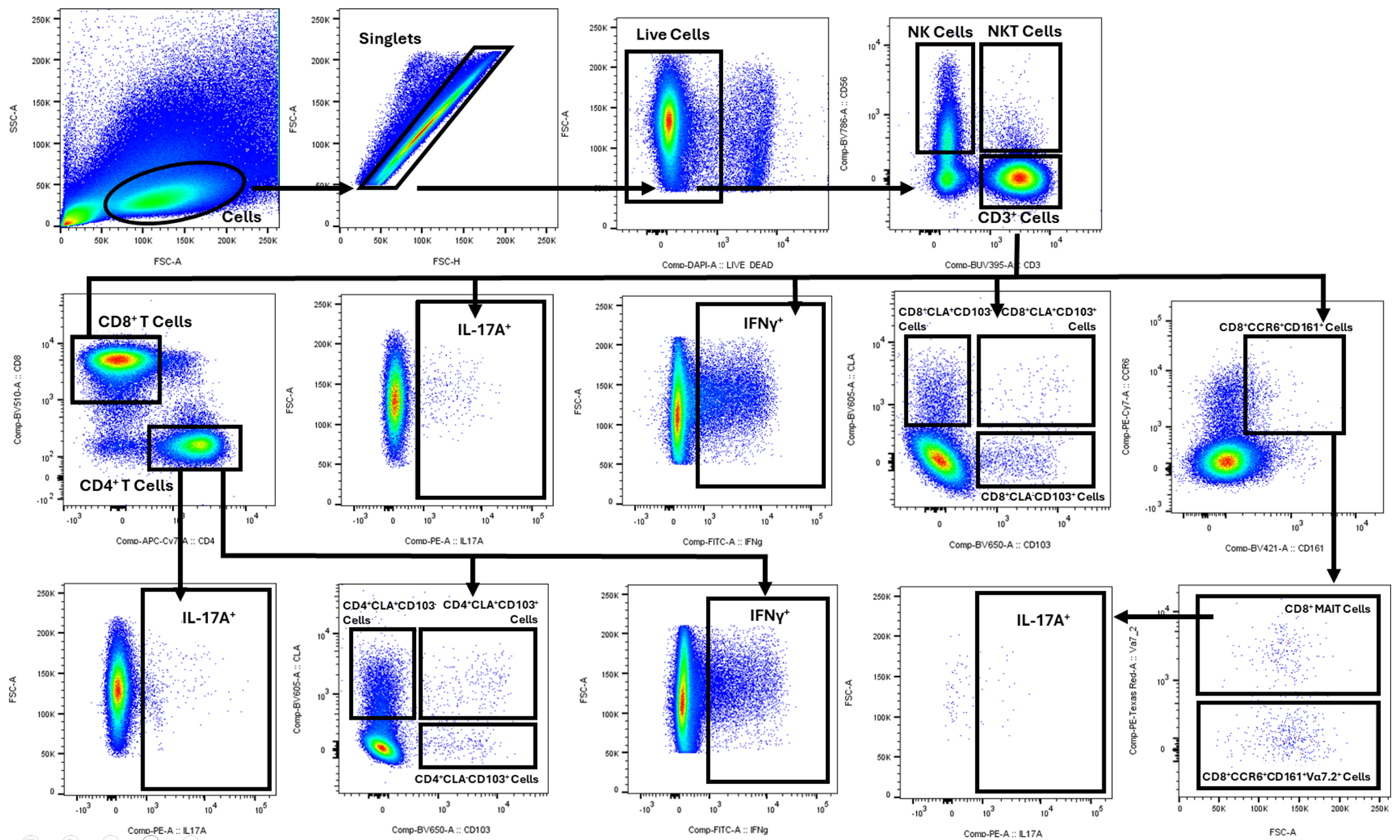
**Supplementary Fig. 1 Flow Cytometry Lymphoid Cell Gating.** Representative gating strategy for Panel B (see Supplementary Table 1), used to identify lymphoid cell populations and assess intracellular cytokine production through flow cytometry. 250,000 live cells were recorded.


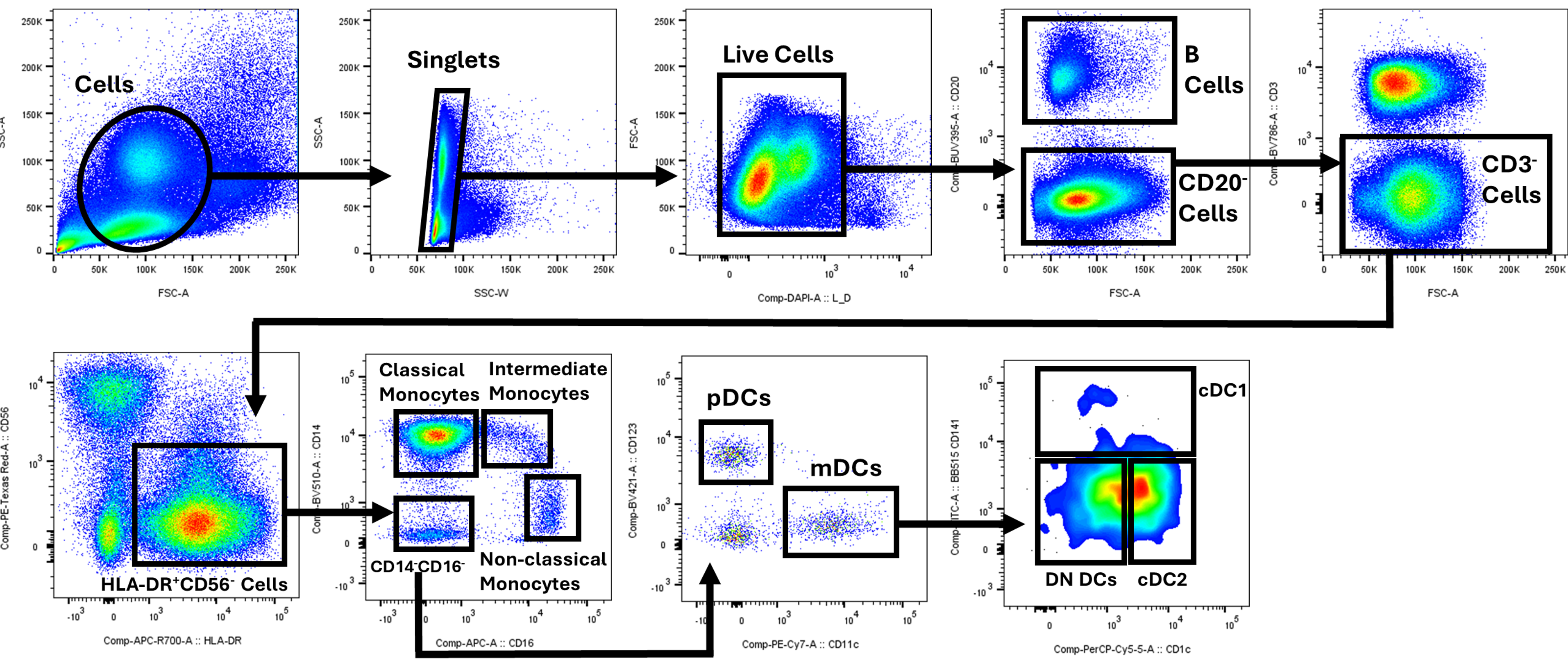


**Supplementary Fig. 2 Flow Cytometry B Cell and Myeloid Cell Gating.** Representative gating strategy for Panel A (see Supplementary Table 1), used to identify B cells and myeloid cell populations through flow cytometry. 250,000 live cells were recorded.


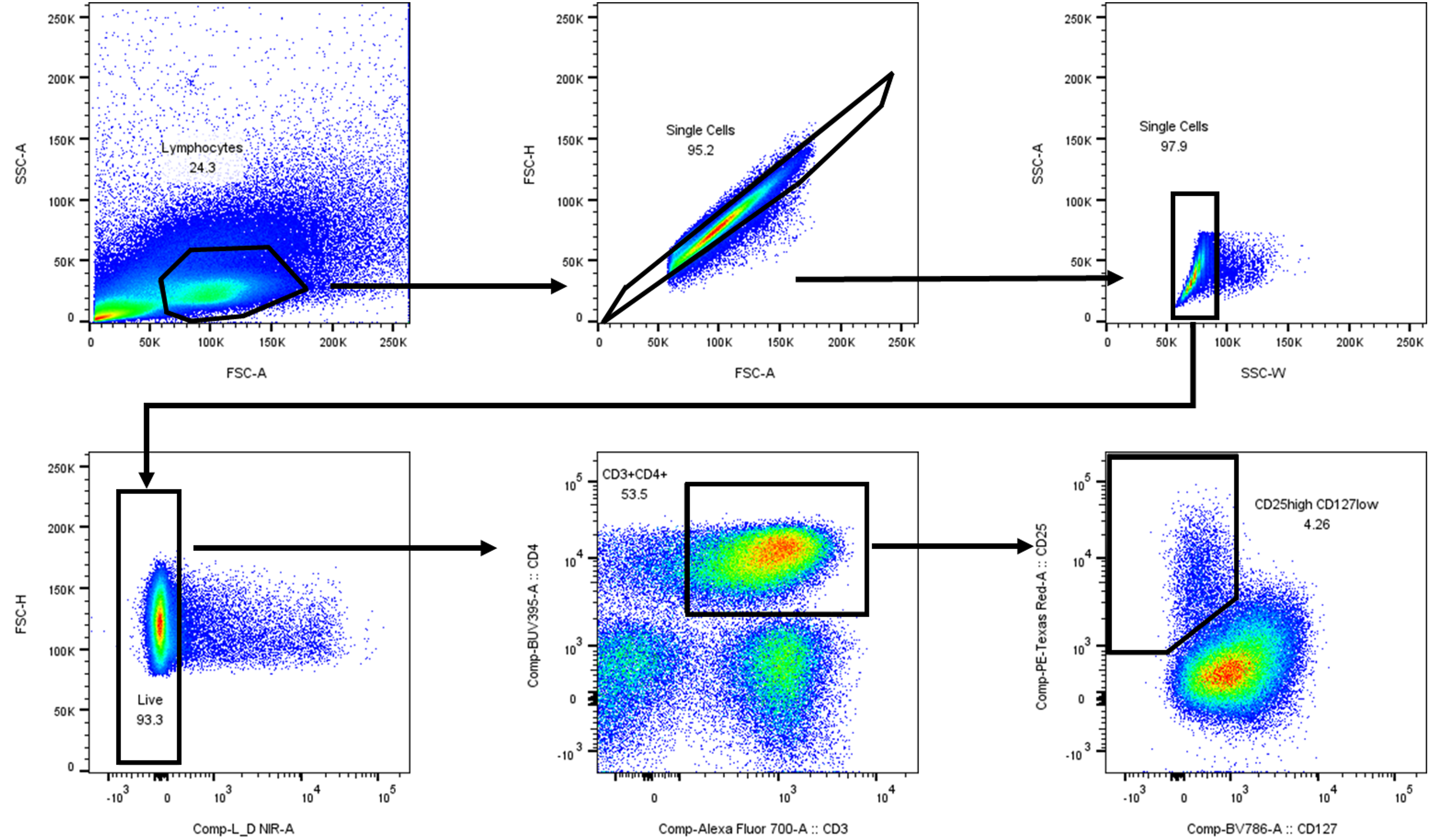


**Supplementary Fig. 3 Flow Cytometry Treg Gating.** Representative gating strategy for Panel C (see Supplementary Table 1), used to identify regulatory T cells through flow cytometry. 250,000 live cells were recorded.

**Host cytotoxic killing capacity as a potential biomarker of therapeutic response to MSCs**

We performed a biomarker assay that predicts clinical response to MSCs, validated in patients with graft-versus-host disease and Crohn disease,^2,3^ in the three patients with psoriasis. This assay is based on the observation that MSCs, upon intravenous infusion, undergo apoptosis induced by recipient cytotoxic cells, and measures the molecular and functional profiles of recipient monocytes exposed to apoptotic MSCs (ApoMSCs). P1 exhibited a borderline positive cytotoxic killing capacity at 11% ApoMSCs; the converse was true for P2 with 30% ApoMSCs. P3 was intermediate with 18% positivity (**Supplementary Fig. 4**).


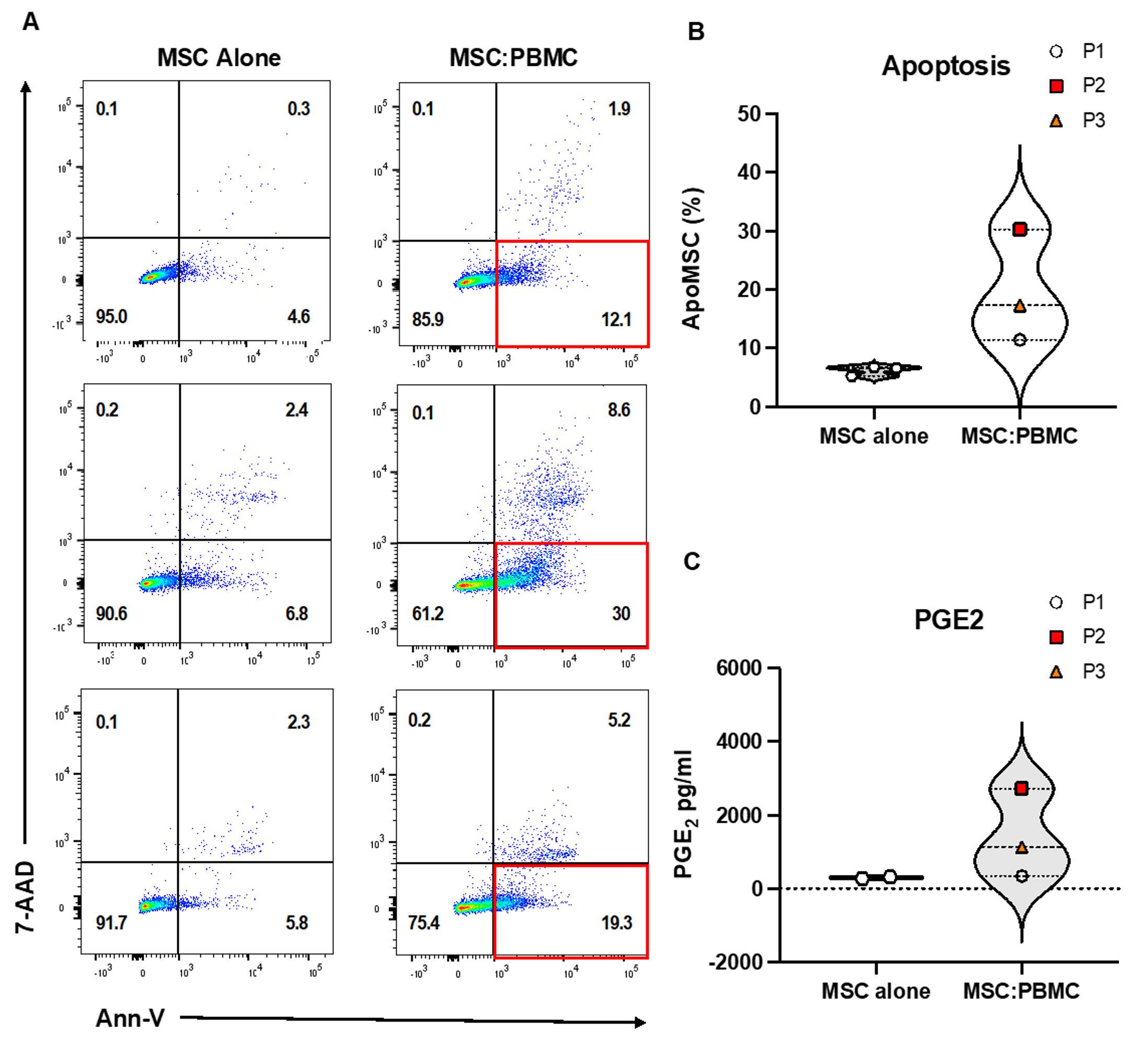


**Supplementary Fig. 4 Apoptotic mesenchymal stromal cells (ApoMSC) and prostaglandin E2 (PGE2) biomarkers were used to predict therapeutic response to MSCs in severe refractory psoriasis patients.** (A) Representative flow cytometry plots displaying the percentage frequency of apoptotic MSCs (ApoMSCs) after co-culture with PBMCs (red squares) derived from 3 psoriasis patients. ApoMSC biomarker was assessed by measuring the percentage of AnnexinV+/7-AAD- MSCs by flow cytometry after 4 hours’ co-culture at a MSC:PBMC ratio of 1:20. MSC alone were used as controls. (B) Violin plot displaying the quantification of apoptosis in controls (MSC alone) and patient samples (MSC:PBMC) revealing variable capacities of three psoriasis patients to induce MSC apoptosis by host immune cells. *P: Patient.* (C) Violin plot displaying quantification of prostaglandin E2 (PGE2) levels detected in the culture supernatant by ELISA after 24 hourss, demonstrating variable levels of PGE2 released through ApoMSCs in three psoriasis patients. P: Patient; Wilcoxon Paired Test between Baseline and Week 1, *p<0.05, ns – non-significant.

**Patients displayed significantly lower frequencies of CD4+ and CD8+ skin-recirculating T cells compared to healthy volunteers (HV) at baseline**

The frequency of CD4+ and CD8+ T cells, B cells and myeloid cell subsets, and key cytokine-producing (IL-17A or IFNγ-producing CD4+ and CD8+) T cells did not differ significantly between psoriasis patients and HV at baseline (**Supplementary Table 3; Supplementary Fig. 5).** However, notable differences were identified in T cell subsets expressing the skin-homing marker cutaneous lymphocyte antigen (CLA) and/or the tissue residency marker CD103. The frequency of CD4+CLA+CD103+ and CD8+CLA+CD103+ skin-recirculating T cells were significantly lower in psoriasis patients compared to HV, however, did not pass FDR multiple corrections testing (p<0.05, FDR>0.05). Similar trends were observed in the CD4+CLA+CD103- and CD8+CLA+CD103- skin-homing T cell counterparts (p<0.05, FDR>0.05). This observation implies the homing of pathogenic T cells in psoriasis skin and subsequent reduction of recirculating counterparts in patients compared to HV.


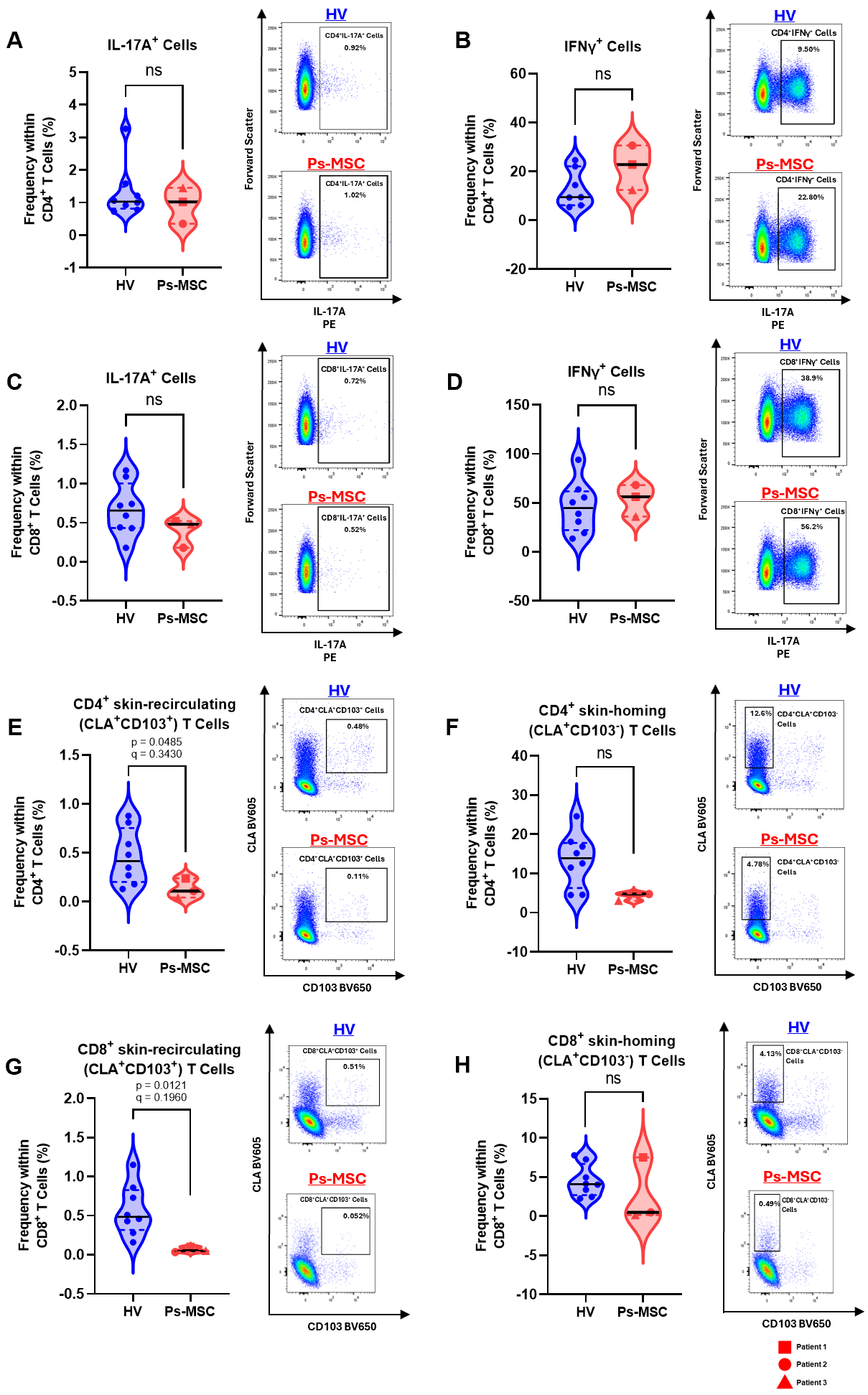


**Supplementary Fig. 5 Frequency of CD4+ and CD8+ T cell subsets in healthy volunteers and psoriasis patients prior to MSC treatment.** Violin plots illustrating the frequency of **A,** CD4^+^IL-17A^+^ T cells, **B,** CD4^+^IFNγ^+^ T cells, **C,** CD8^+^IL-17A^+^ T cells, **D,** CD8^+^IFNγ^+^ T cells, **E**, CD4^+^CLA^+^CD103^+^ T cells, **F**, CD4^+^CLA^+^CD103^-^ T cells, **G**, CD8^+^CLA^+^CD103^+^ T cells and **H**, CD8^+^CLA^+^CD103^+^ T cells, expressed either as a percentage of the parent macro-population or within live cells, in healthy volunteers (n=8) and psoriasis patients (n=3), with representative flow cytometry plots (right). *Mann-Whitney Test and FDR multiple comparisons test. *p<0.05, ns – non-significant. FDR q<0.05.***FIG 3.** Three psoriasis patients display different immunophenotypes. Line graphs illustrating the frequency of **A,** CD4^+^IL-17A^+^ T cells, **B,** CD4^+^IFNγ^+^ T cells, **C,** CD8^+^IL-17A^+^ T cells and **D,** CD8^+^IFNγ^+^ T cells, as a percentage of the parent macro-population, before (baseline) and after 2 infusions with UC-MSCs a week apart in psoriasis patients.  *Red arrows indicate the time of MSC infusions. Grey arrows denote the duration of biologic/biosimilar or when it was paused or reinitiated. W: Week; Wilcoxon Paired Test between Baseline and Week 1, *p<0.05, ns – non-significant.*

**
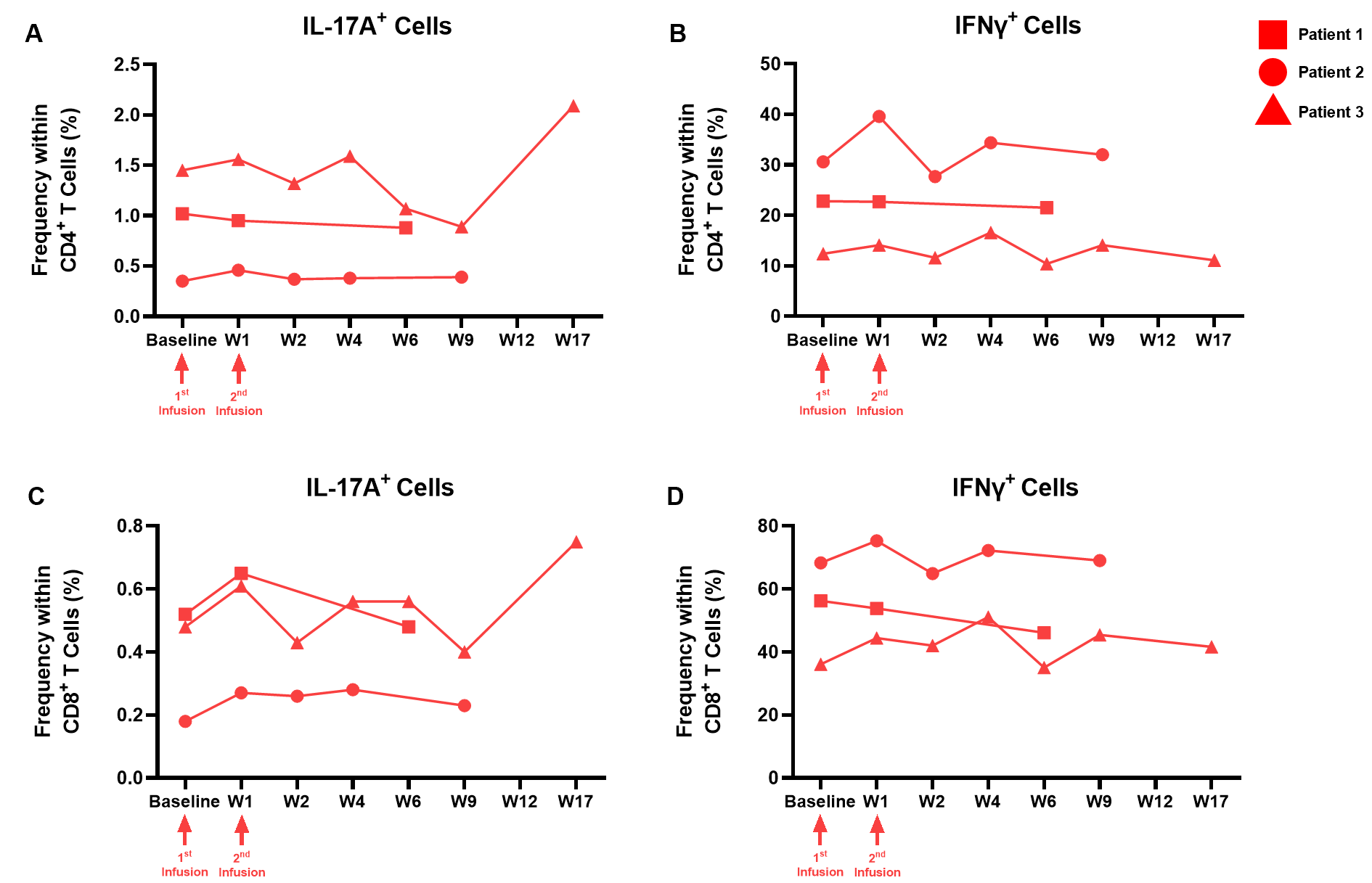
**

**Supplementary Fig. 6 Frequency of IL-17A^+^ vs IFNꝩ^+^ CD4^+^ and CD8^+^ T cells over time in the three psoriasis patients demonstrate their different immune profiles.** Line graphs illustrating the frequency of **A**, CD4^+^IL-17A^+^ T cells; **B**, CD8^+^IL-17A^+^ T cells; **C**, CD4^+^IFNꝩ^+^ T cells and **D**, CD8^+^IFNꝩ^+^ T cells, expressed as a percentage within parent populations before and after UC-MSC infusions. *W: Week.*

**Peripheral blood monocytes switch from CD14^+^CD16^+^ intermediate to CD14^Lo^CD16^+^ non-classical phenotype following UC-MSCs**

Changes in frequency of monocyte subsets also occurred (**Supplementary Fig. 7**). Reduction in frequency of CD14^+^CD16^+^ intermediate monocytes corresponded to reduction in PASI in all patients. The converse was observed for CD14^Lo^CD16^+^ non-classical monocytes where increased frequency tended to correspond with clinical improvement.

**
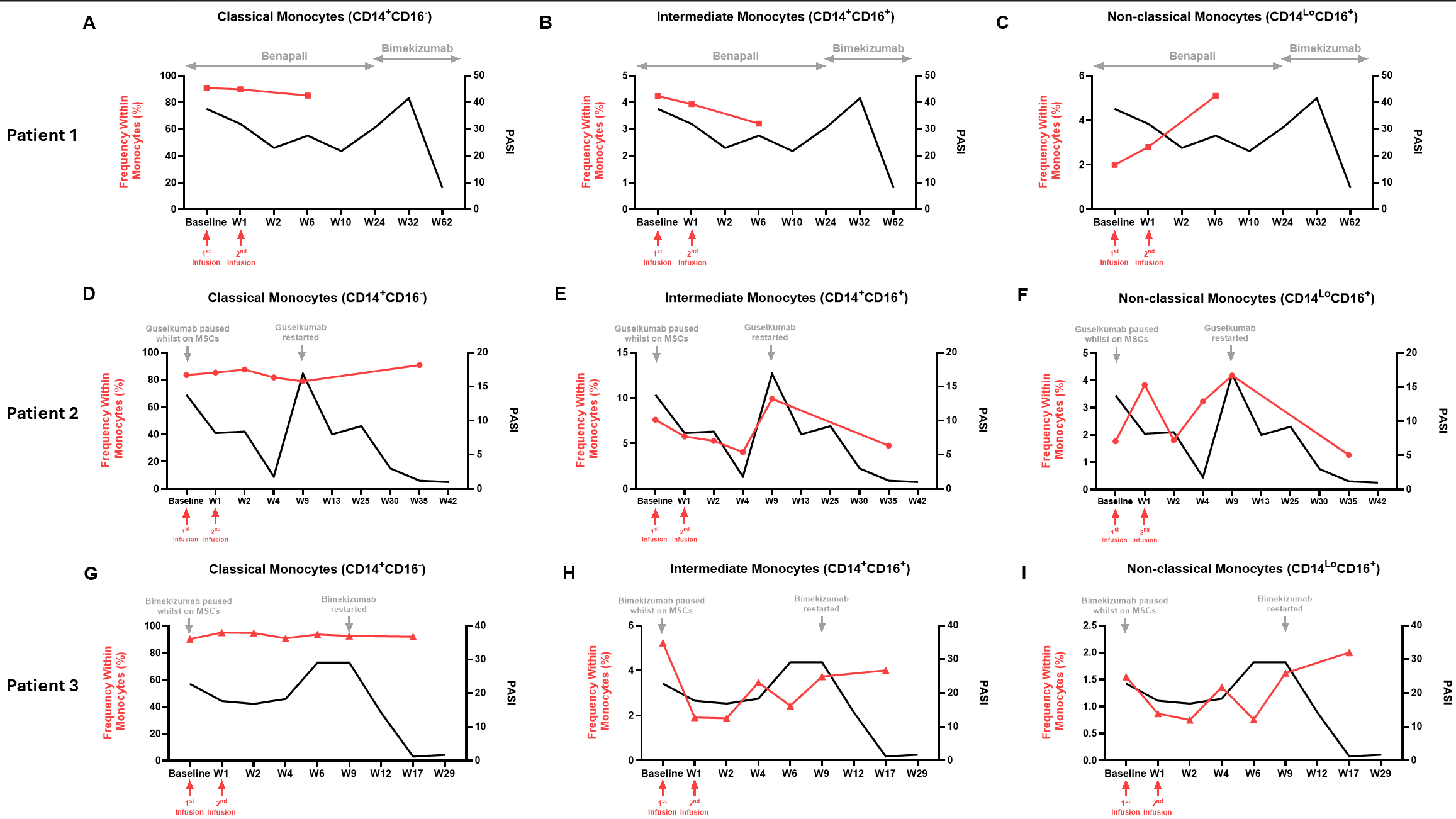
**

**Supplementary Fig. 7 Frequency of monocyte subsets in peripheral blood psoriasis patients before and after MSC therapy.** Line graphs illustrating the frequency of CD14^+^CD16^-^ classical, CD14^+^CD16^+^ intermediate and CD14^Lo^CD16^+^ non-classical monocytes in peripheral blood as a percentage within the monocyte macro-population (*red line*), superimposed with the PASI score (*black line*), before and after MSC infusions in (A-C) Patient 1, (D-F) Patient 2 and (G-I) Patient 3. *Red arrows indicate the time of MSC infusions. Grey arrows denote the duration of biologic/biosimilar or when it was paused or reinitiated. W: Week.*

*
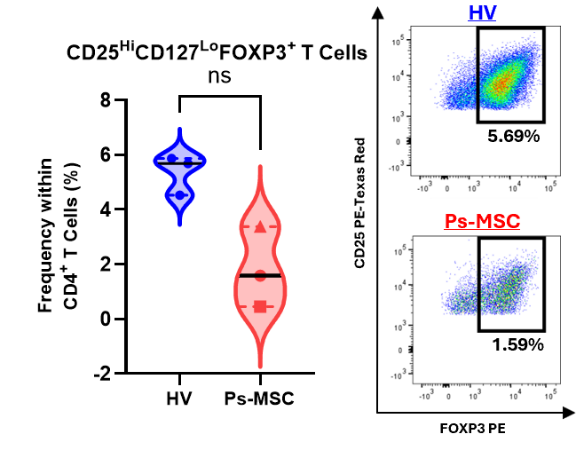
***Supplementary Fig. 8 Frequency of regulatory T cells (Tregs) in psoriasis patients at baseline compared to healthy volunteers (HV).** (A) Violin plot illustrating the frequency of Tregs, defined as % frequency within CD4+ T cells in HV (n=3) and psoriasis patients before UC-MSC infusion (n=3). Representative flow cytometry plots are displayed on the right. Mann-Whitney Test and FDR multiple comparisons test. ns – non-significant. FDR q<0.05.

**Reference**

1. Andres-Ejarque R, Ale HB, Grys K, Tosi I, Solanky S, Ainali C et al. Enhanced NF-κB signaling in type 2 dendritic cells at baseline predicts non-response to adalimumab in psoriasis. *Nat Commun.* 2021; **12**:4741.
2. Galleu A, Riffo-Vasquez Y, Trento C, Lomas C, Dolcetti L, Cheung TS et al. Apoptosis in mesenchymal stromal cells induces in vivo recipient-mediated immunomodulation. *Sci Transl Med.* 2017; **9**:eaam7828.
3. Cheung TS, Giacomini C, Cereda M, Avivar-Valderas A, Capece D, Bertolino GM et al. Apoptosis in mesenchymal stromal cells activates an immunosuppressive secretome predicting clinical response in Crohn’s disease. *Mol Ther.* 2023; **31**:3531- 3544.
