# Supplementary figures and images for: Mesenchymal stromal cells as rescue therapy in biologic-refractory psoriasis: insights from a case series
Source: Front Immunol. 2025 Sep 3;16:1656724. doi: 10.3389/fimmu.2025.1656724 (PMC12440939; doi:10.3389/fimmu.2025.1656724)

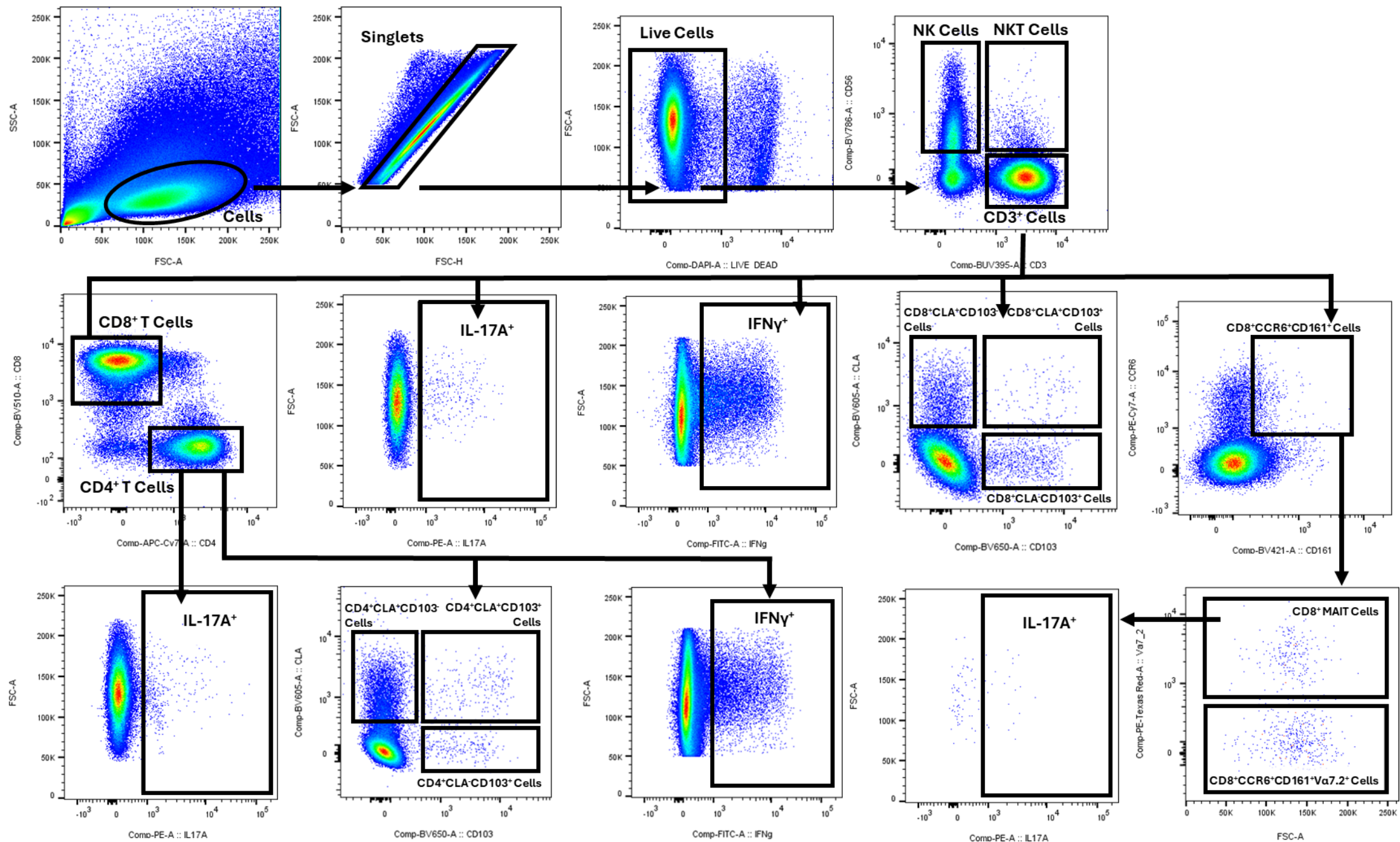

Supplement: Supplementary file 2 [file Image1.pdf]

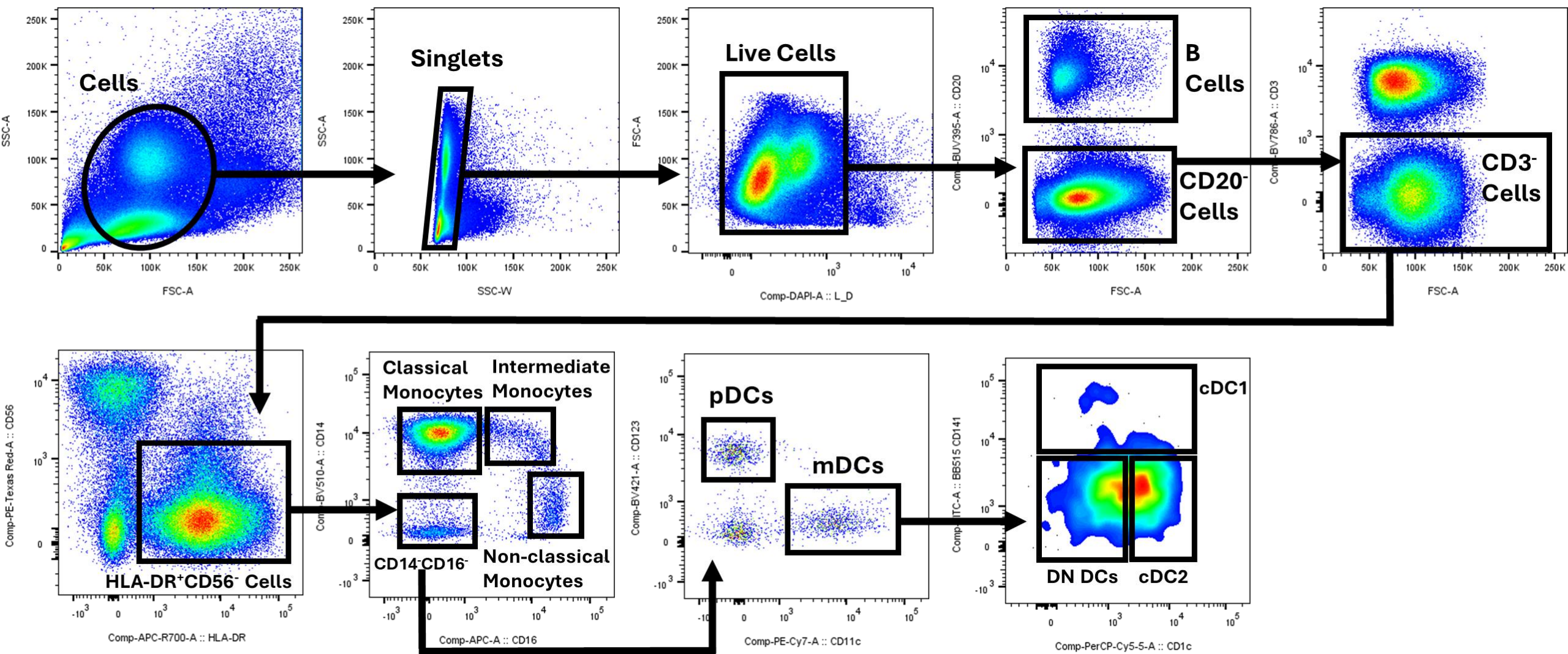

Supplement: Supplementary file 3 [file Image2.pdf]

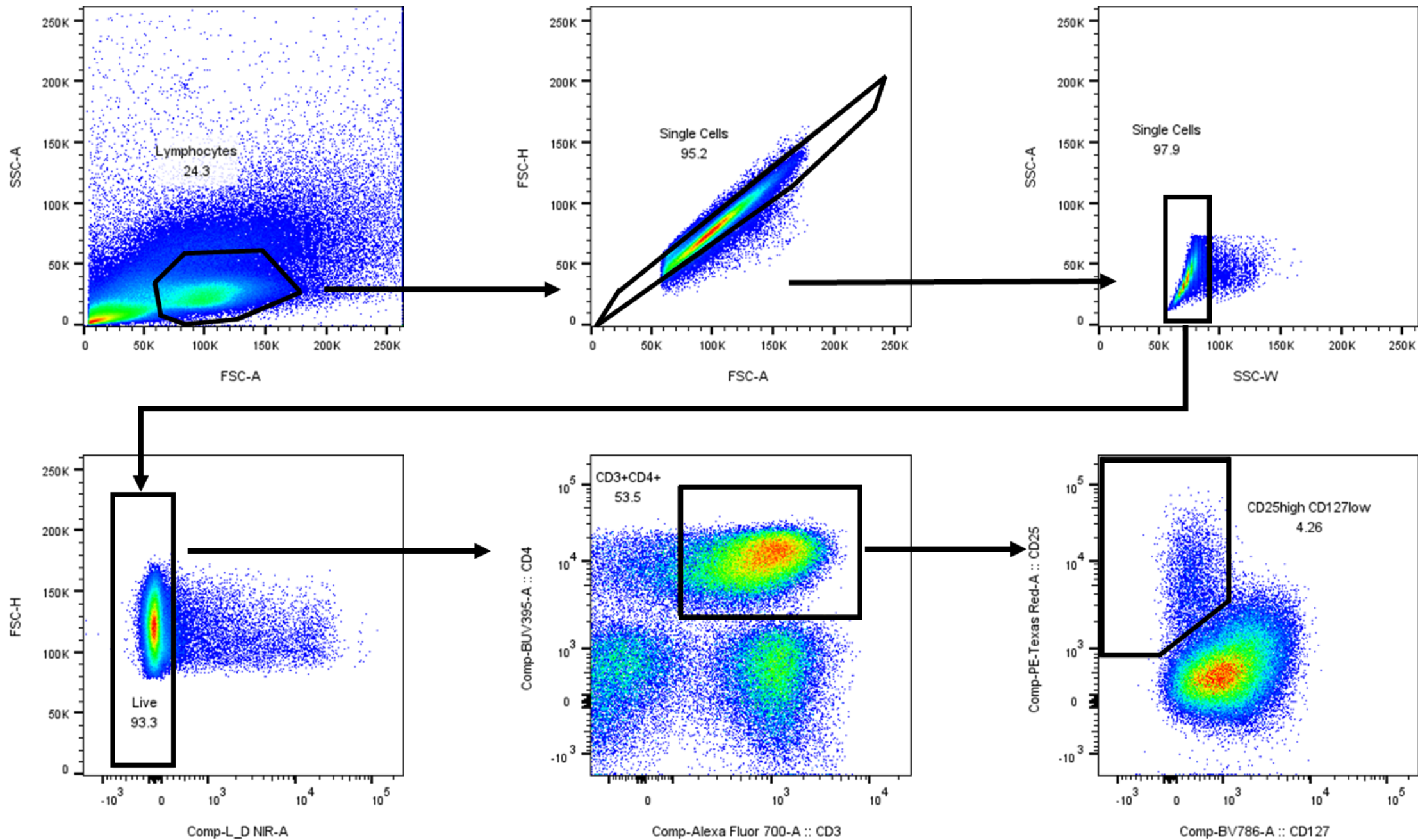

Supplement: Supplementary file 4 [file Image3.pdf]

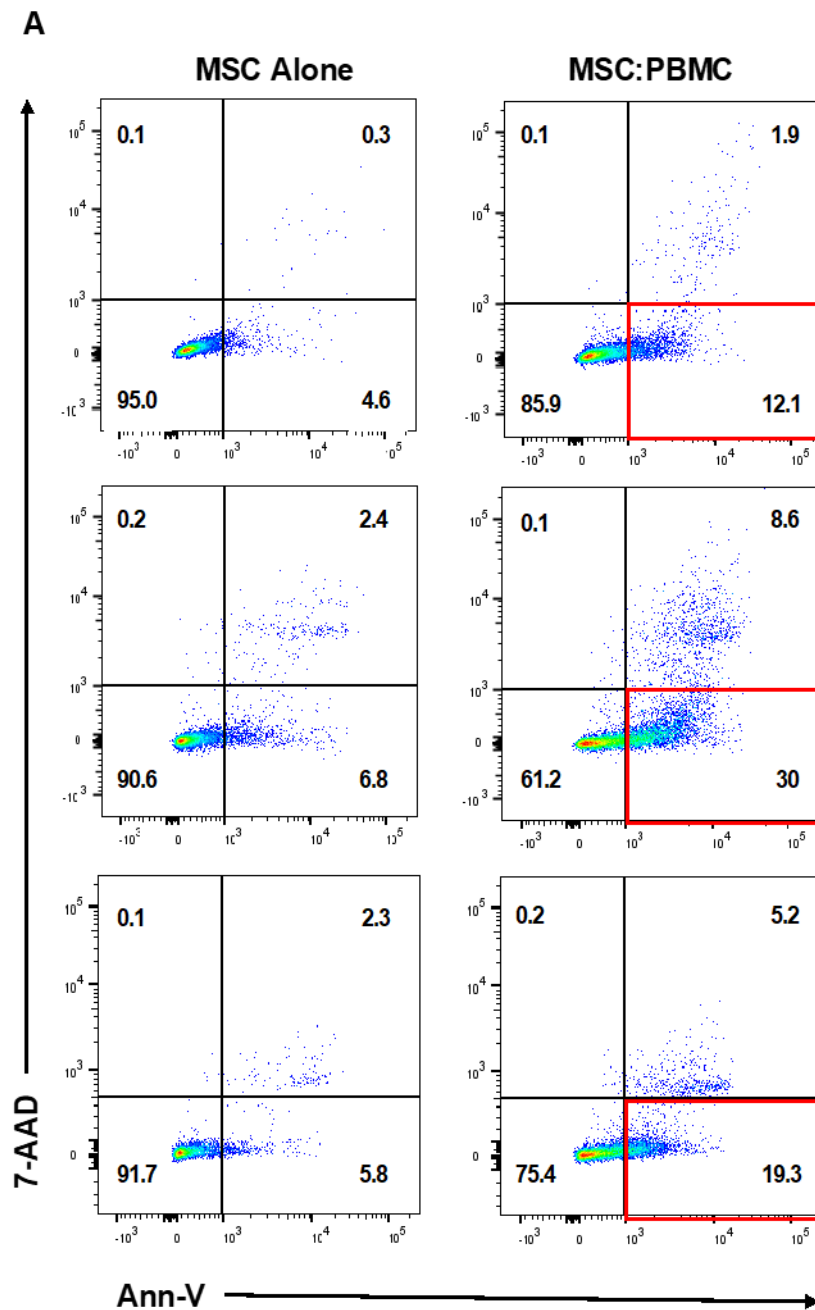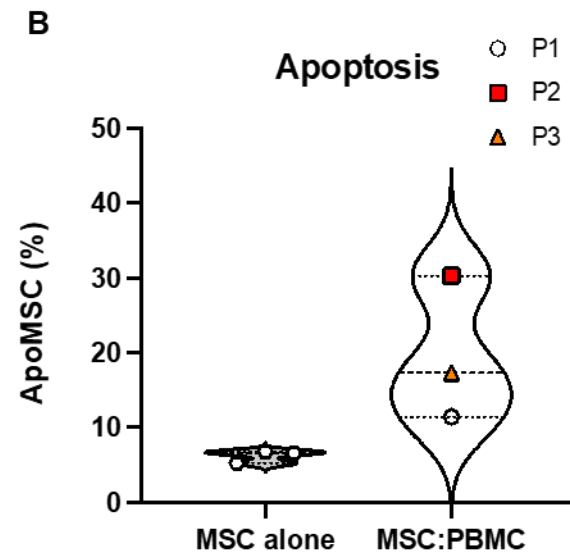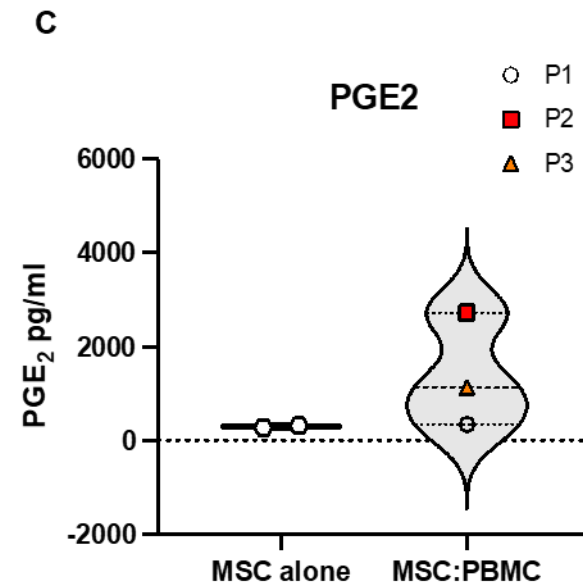

Supplement: Supplementary file 5 [file Image4.pdf]

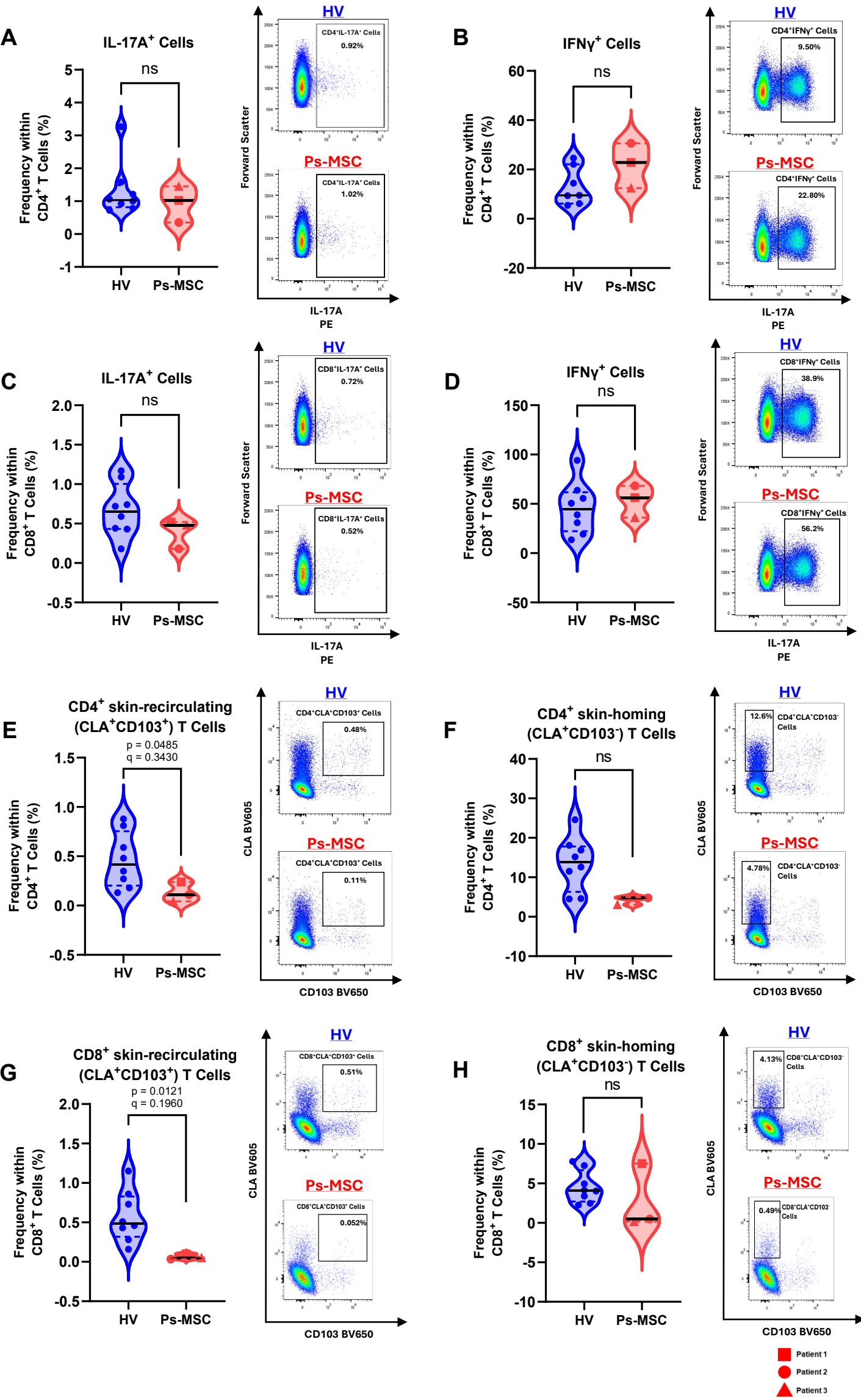

Supplement: Supplementary file 6 [file Image5.pdf]

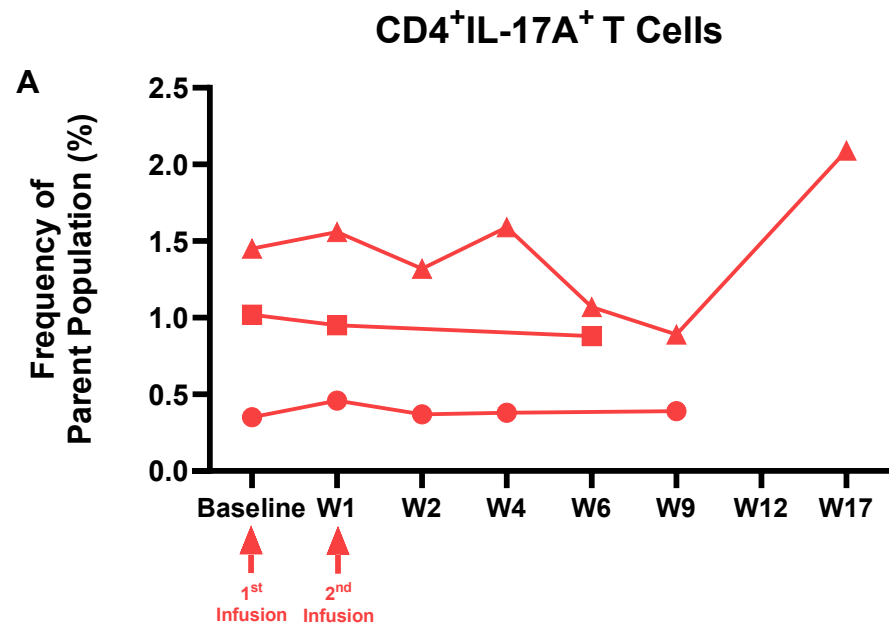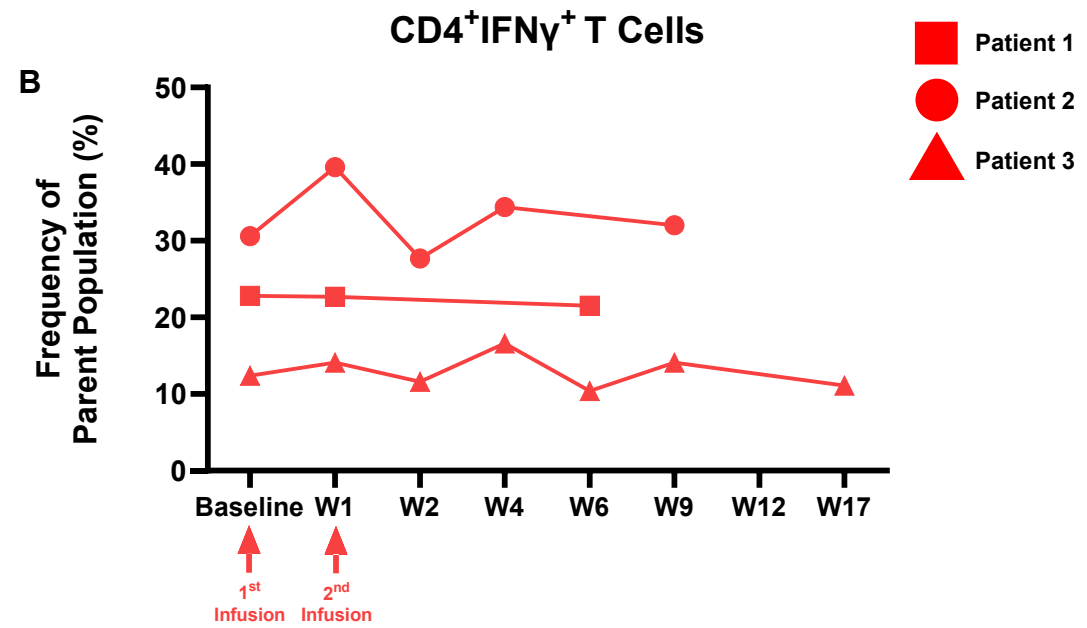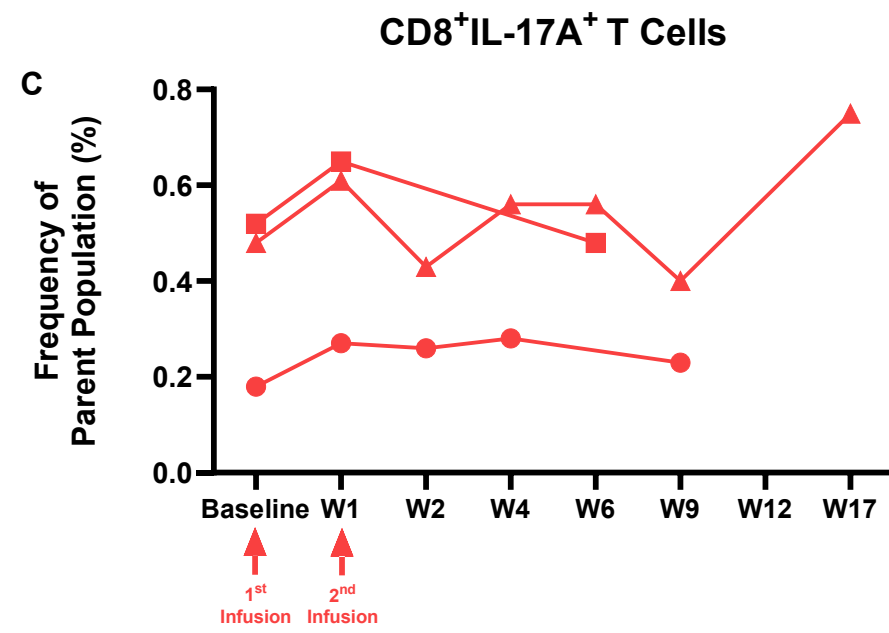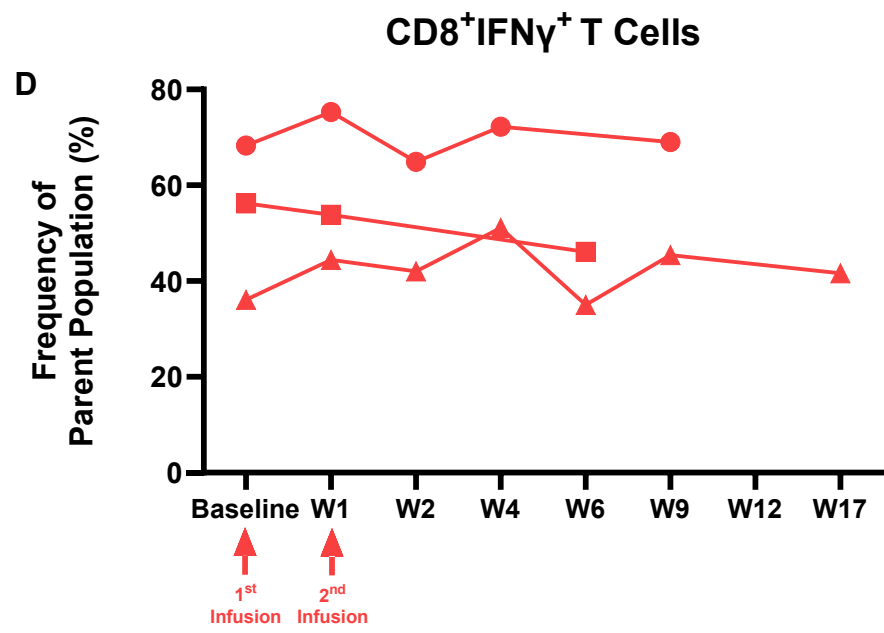

Supplement: Supplementary file 7 [file Image6.pdf]

Patient 1

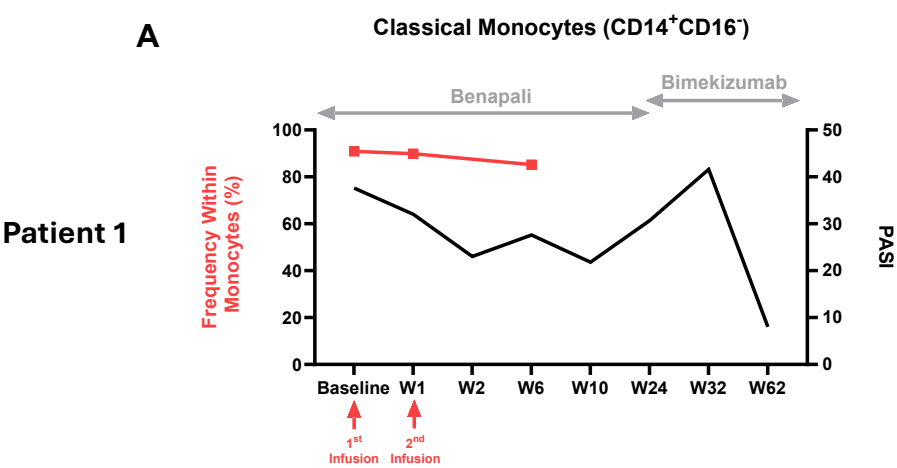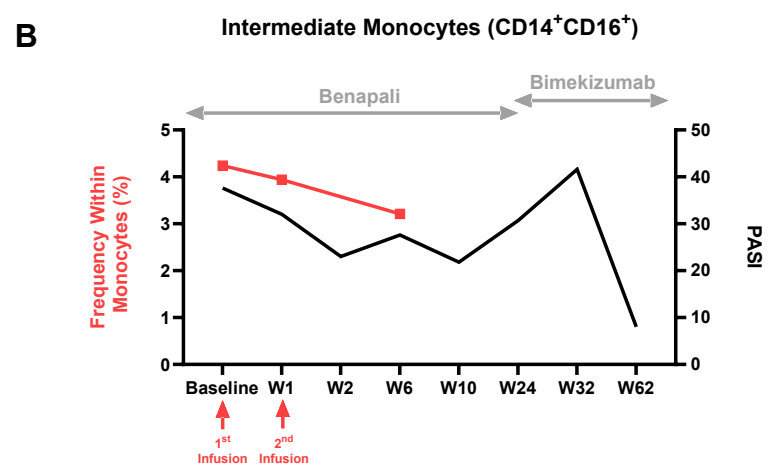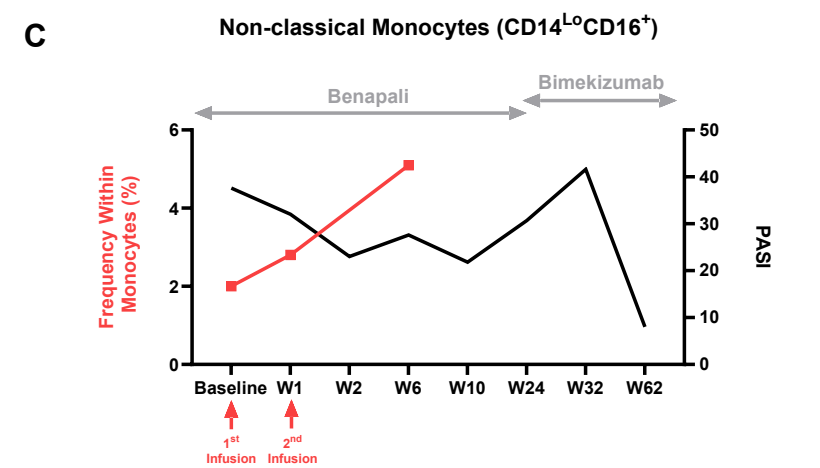

Patient 2

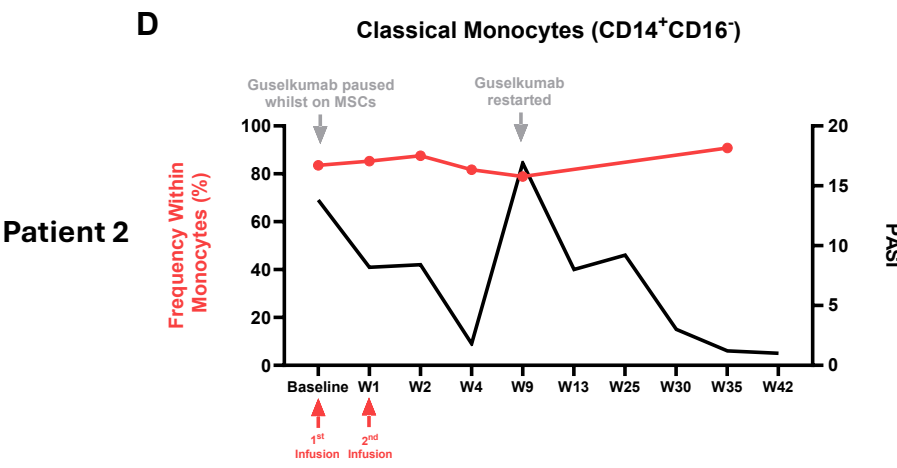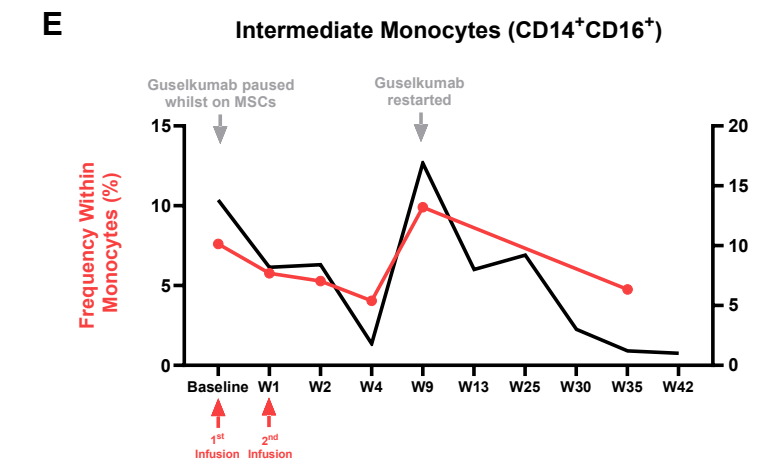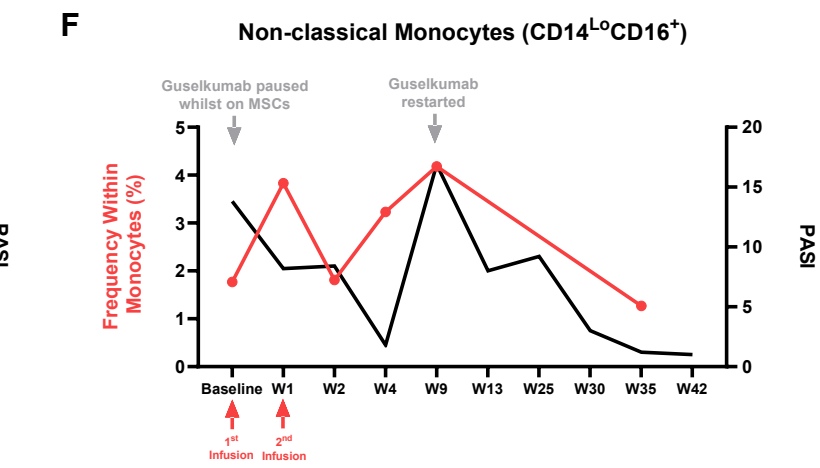

Patient 3

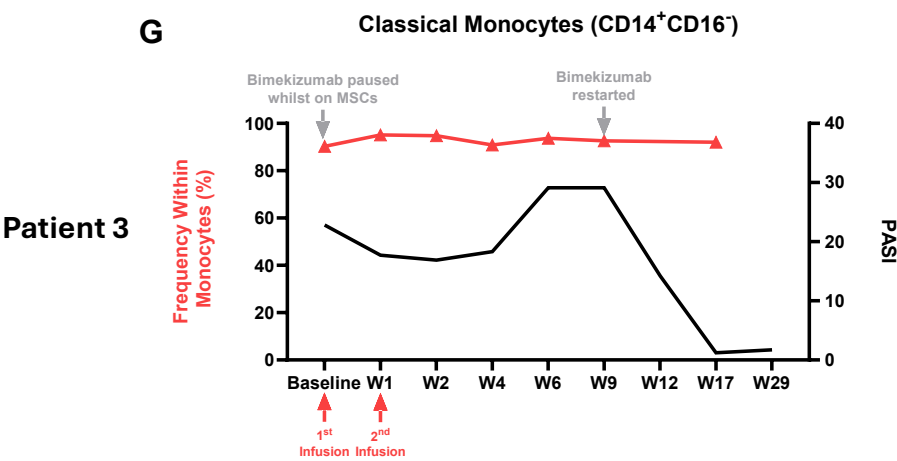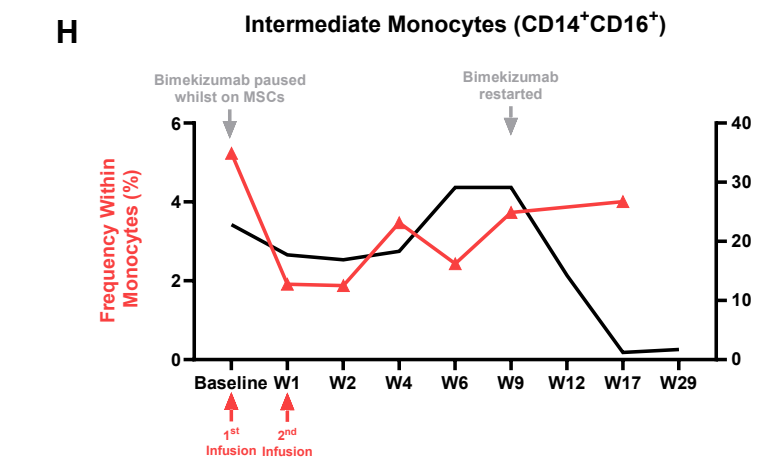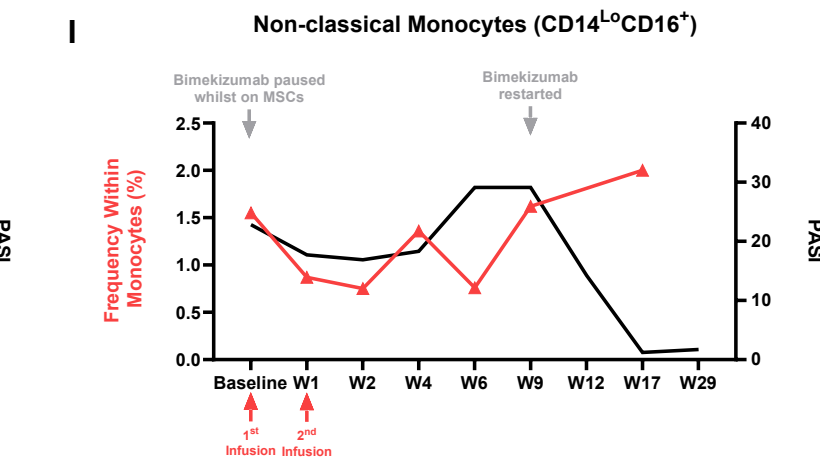

Supplement: Supplementary file 8 [file Image7.pdf]

CD25<sup>Hi</sup>CD127<sup>Lo</sup>FOXP3<sup>+</sup> T Cells

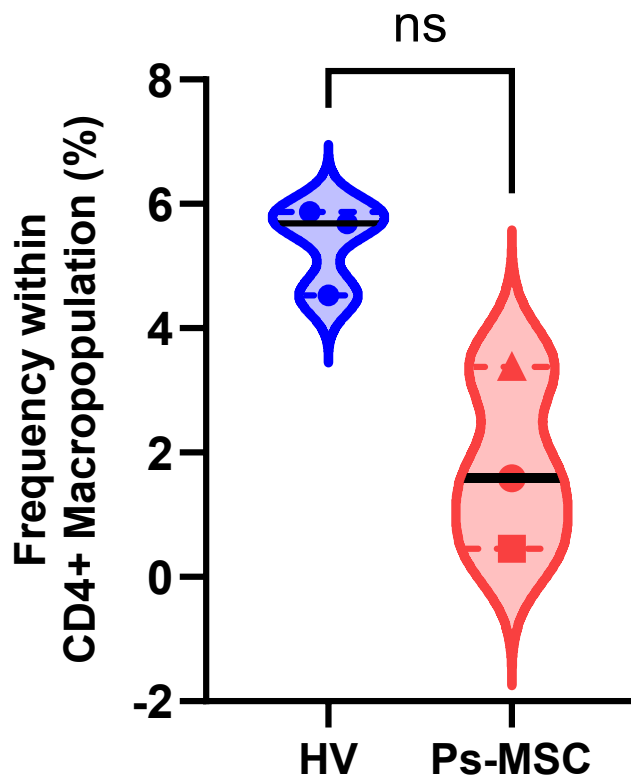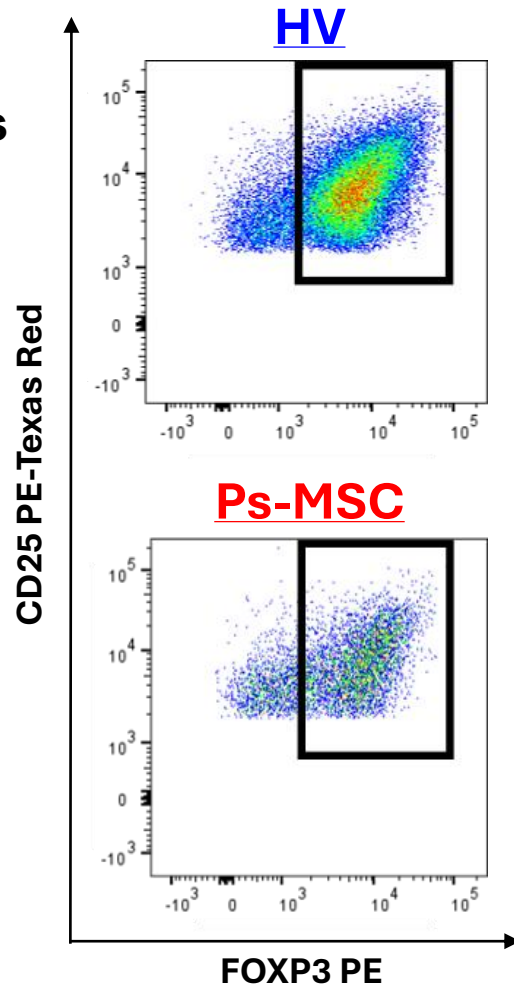

Supplement: Supplementary file 9 [file Image8.pdf]
